# Neocentromeres Form Efficiently at Multiple Possible Loci in *Candida albicans*

**DOI:** 10.1371/journal.pgen.1000400

**Published:** 2009-03-06

**Authors:** Carrie Ketel, Helen S. W. Wang, Mark McClellan, Kelly Bouchonville, Anna Selmecki, Tamar Lahav, Maryam Gerami-Nejad, Judith Berman

**Affiliations:** 1 Department of Genetics, Cell Biology, and Development, University of Minnesota, Minneapolis, Minnesota, United States of America; 2 Department of Microbiology, University of Minnesota, Minneapolis, Minnesota, United States of America; The University of North Carolina at Chapel Hill, United States of America

## Abstract

Centromeres are critically important for chromosome stability and integrity. Most eukaryotes have regional centromeres that include long tracts of repetitive DNA packaged into pericentric heterochromatin. Neocentromeres, new sites of functional kinetochore assembly, can form at ectopic loci because no DNA sequence is strictly required for assembly of a functional kinetochore. In humans, neocentromeres often arise in cells with gross chromosome rearrangements that rescue an acentric chromosome. Here, we studied the properties of centromeres in *Candida albicans*, the most prevalent fungal pathogen of humans, which has small regional centromeres that lack pericentric heterochromatin. We functionally delimited centromere DNA on Chromosome 5 (*CEN5*) and then replaced the entire region with the counter-selectable *URA3* gene or other marker genes. All of the resulting *cen5Δ::URA3* transformants stably retained both copies of Chr5, indicating that a functional neocentromere had assembled efficiently on the homolog lacking *CEN5* DNA. Strains selected to maintain only the *cen5Δ::URA3* homolog and no wild-type Chr5 homolog also grew well, indicating that neocentromere function is independent of the presence of any wild-type *CEN5* DNA. Two classes of neocentromere (neoCEN) strains were distinguishable: “proximal neoCEN” and “distal neoCEN” strains. Neocentromeres in the distal neoCEN strains formed at loci about 200–450 kb from *cen5Δ::URA3* on either chromosome arm, as detected by massively parallel sequencing of DNA isolated by CENP-A^Cse4p^ chromatin immunoprecipitation (ChIP). In the proximal neoCEN strains, the neocentromeres formed directly adjacent to *cen5Δ::URA3* and moved onto the *URA3* DNA, resulting in silencing of its expression. Functional neocentromeres form efficiently at several possible loci that share properties of low gene density and flanking repeated DNA sequences. Subsequently, neocentromeres can move locally, which can be detected by silencing of an adjacent *URA3* gene, or can relocate to entirely different regions of the chromosome. The ability to select for neocentromere formation and movement in *C. albicans* permits mechanistic analysis of the assembly and maintenance of a regional centromere.

## Author Summary

Centromere function is essential for proper chromosomal segregation. Most organisms, including humans, have regional centromeres in which centromere function is not strictly dependent on DNA sequence. Upon alteration of chromosomes, new functional centromeres (neocentromeres) can form at ectopic positions. The mechanisms of neocentromere formation are not understood, primarily because neocentromere formation is rarely detected. Here. we show that *C. albicans*, an important fungal pathogen of humans, has small regional centromeres and can form neocentromeres very efficiently when normal centromere DNA is deleted, and the resulting chromosomes are stably propagated. Neocentromeres can form either very close to the position of the deleted centromere or at other positions along the chromosome arms, including at the telomeres. Subsequently, neocentromeres can move to new chromosomal positions, and this movement can be detected by silencing of a counterselectable gene. The features common to sites of neocentromere formation are longer-than-average intergenic regions and the proximity of inverted or direct repeat sequences. The ability to select for neocentromere formation and movement in *C. albicans* permits mechanistic analysis of the assembly and maintenance of a regional centromere.

## Introduction

Centromeres, the DNA regions at which microtubules attach to and segregate daughter chromosomes, are essential for genome integrity. Point centromeres have been characterized extensively because of their small size and relative simplicity, especially in *Saccharomyces cerevisiae*. They are composed of one centromere-specific nucleosome that spans <200 bp of DNA, organized in a tripartite structure that includes a specific DNA binding site necessary for centromere function (reviewed in [1],[2]). In contrast, regional centromeres are found in most eukaryotes, including fungi other than a subgroup of the Saccharomycotina [3]. They span very large DNA domains (10′s to 1000′s of kb) and are organized into core DNA regions associated with centromere-specific nucleosomes and flanked by highly repetitive DNA packaged into pericentric heterochromatin (reviewed in [4]). Regional centromere function is epigenetic in character [5]–[8]: rather than being dependent upon a specific DNA sequence, the presence of CENP-A, the centromere-specific histone H3 variant, defines the position of a functional centromere. It is thought that CENP-A is regulated by its stabilization at functional centromeres/kinetochores, because at non-centromeric loci CENP-A is removed and proteolyzed [9], (reviewed in [7]).

Neocentromeres, defined as functional kinetochores that assemble at ectopic positions, usually appear together with other chromosome rearrangement events (reviewed in [6]). Neocentromeres have the properties of active centromeres, and, by definition, they associate with CENP-A [10]–[12]. Over 90 examples of human neocentromeres have been documented, most involving the formation of supernumerary chromosomes and often associated with developmental disabilities or specific cancers [6]. Many more neocentromeres likely escape detection because they are eliminated during development [6],[8],[13]. It is not known if neocentromere formation occurs first, followed by mutation of the natural centromere or, conversely, if mutation of the natural centromere leads to neocentromere formation.

The chromosomal position of neocentromeres is different in different organisms. In Drosophila, they have been found only adjacent to chromosome breaks that inactivated the original centromere [14],[15]. In *S. pombe* they appear only at telomeric loci [16]. In contrast, human neocentromeres exhibit flexible adaptation to changes in chromosome structure, often appearing far from the site of the original centromere at either terminal or submetacentric loci (reviewed in [6]).

No specific DNA sequence properties necessary for functional regional centromere assembly have been identified. Regional centromeres in humans, flies, plants and fungi are composed of long tracts of repetitive DNA, yet repeat tracts are not absolutely required for centromere function or for the formation of neocentromeres (reviewed in [8], [17]–[19]). In *S. pombe*, pericentric heterochromatin formation is necessary for efficient *de novo* assembly of a functional kinetochore [20], as well as for formation of telocentric neocentromeres [16].

*C. albicans*, an opportunistic fungal pathogen that resides as a commensal in its human host, possess regional centromeres that are much smaller and simpler than other regional centromeres [3],[21]. Each of its 8 diploid chromosomes has a centromere that is regional based on its size (∼3–4.5 kb that specifically associates with CENP-A^Cse4p^ [22],[23]), the lack of tripartite point-centromere DNA structure, the presence of several orthologs of proteins found only at regional centromeres, and the absence of orthologs of proteins found only at point centromeres [3].

Most notably, *C. albicans* CENs lack pericentric heterochromatin: CENP-A associated core sequences are not embedded in long tracts of repetitive DNA [21],[22]; there are no clear orthologs of either heterochromatin protein 1 (HP1) or of enzymes necessary for the methylation of histone H3 lysine 9; and there is no homolog of CENP-V, a protein that regulates the extent of pericentric chromatin in human cells [24]. Consistent with a lack of pericentric heterochromatin, genes near the centromeres are transcribed at levels close to the average level of transcription across the genome (K. E. S. Tang and JB, data not shown). Furthermore periodic nucleosome spacing is seen at inactive centromere DNA and not at active CENs, suggesting that nucleosomes at active CENs do not associate tightly with a specific DNA sequence [21]. Naked *CEN7* DNA used to transform *C. albicans* did not permit *de novo* assembly of centromere function [21],[22]. Taken together, these observations suggest that, like other regional CENs, the assembly of a centromere and the inheritance of centromere function in *C. albicans* requires epigenetic properties conferred by the association of CENP-A and other kinetochore proteins, rather than by a specific DNA sequence.

The stoichiometry of microtubules and centromere-specific nucleosomes differs in different organisms. *S. cerevisiae* has only one CENP-A nucleosome and one microtubule per centromere, while *S. pombe* has ∼2–3 CENP-A nucleosomes [25] and ∼2–4 microtubules per centromere [26]. This suggests that one microtubule is attached to kinetochore proteins assembled at each CENP-A nucleosome [25]. In humans, the number of CENP-A nucleosomes is thought to be far larger than the number of microtubule attachments [4],[27]. In *C. albicans* there are ∼8 CENP-A^Cse4p^ molecules per centromere, presumed to be organized into 4 centromere-specific nucleosomes, and only one microtubule per centromere [25]. This suggests that only one of the four CENP-A nucleosomes at each centromere assembles a kinetochore structure that binds a microtubule. Thus, the prevailing model is that at *C. albicans* CENs, as at human centromeres, some CENP-A-containing nucleosomes bind microtubules while others do not.

Seven of the eight *C. albicans* centromeres are near short repeats; only the centromere of Chr7 (*CEN7*) does not have obvious repeat sequences nearby [22]. Yet most analysis of *C. albicans* centromere function has been performed with *CEN7* DNA, which is necessary for Chr7 stability [23]. On Chrs 2, 3 and 6 there are direct repeats within ∼3 kb of the centromere core sequence. On Chrs 1, 4, 5 and R the CENP-A bound centromere core DNA is flanked by a short inverted repeat (IR). The palindromic structure of the four centromeres with a flanking IR is most reminiscent of the structure of *S. pombe* and other regional centromeres.

Here, we studied the properties of *C. albicans CEN5*, a centromere with a palindromic structure, by replacing the *CEN5* DNA and the flanking IR with a *URA3* marker. We found that it behaved like a regional centromere: the resulting *cen5Δ::URA3* Chr5 derivatives were stably maintained through mitosis by efficient formation of a neocentromere at one of several non-centromeric loci. Loss of the wild-type chromosome 5 homolog and homozygosis of the *neoCEN5* homolog demonstrated that cells can survive in the absence of any *CEN5* DNA. Some of the *cen5Δ* transformants formed a “proximal neoCEN” near *cen5Δ::URA3*, which subsequently moved onto, and silenced the *URA3* gene. Other *cen5Δ* transformants formed “distal neoCENs” at several different loci far from the deleted *CEN5* locus. Thus, neocentromere formation does not require a specific DNA sequence and can occur at several different chromosomal loci.

## Results

### *CEN5* Function Resides within a Central Core CENP-A Binding Site Flanked by Inverted Repeats

**Figure 1:**
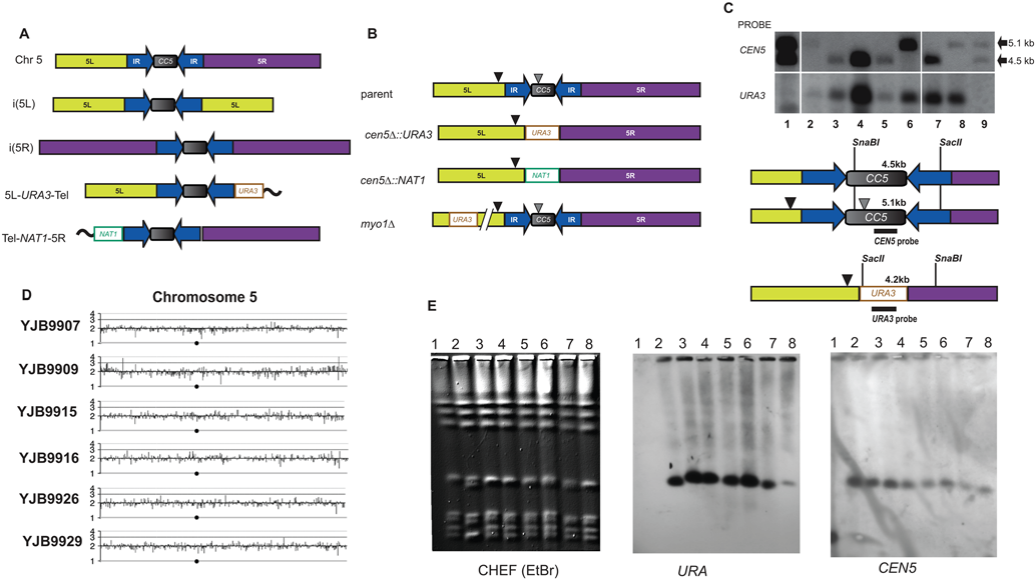
Deletion analysis of *Candida albicans CEN*5. A. Central core and flanking inverted repeat define the Chr5 region with centromere function. *C. albicans CEN5* is composed of a central core (*CC5*, gray rectangle, a flanking inverted repeat (IR, blue arrows), and adjacent left (yellow, 5L) and right (purple, 5R) chromosome arms. Isochromosomes i(5L) and i(5R), derived from wild-type Chr5 (top), arose in strains YJB9891 and YJB8287, respectively. Loss rates of i(5L) are ∼10^−5^ loss events/division. Telomere truncation derivatives 5L-*URA3*-Tel (YJB9772, loss rate ∼10^−3^ events/division) and Tel-*NAT1*-5R (YJB9858, loss rate ∼10^−4^ events/division) were generated by deletion of the right or left arm of Chr5 within ∼0.5 kb of the inverted repeat. *CC5* and the flanking IR is the only region common to these stable derivatives, indicating that centromere function resides within this region. Illustrations are not to scale. B. Replacement of *CEN5*. Wild-type cells carry two homologs of *CEN5:* the long homolog contains two LTRs (*psi* within *CC5* and *episemon* to the left of *CEN5* (triangles)) that are absent in the short homolog. *CEN5* was replaced by *URA3* or *NAT1*, which inserted at similar frequencies into the two *CEN5* homologs. Transformation frequencies were similar for *cen5Δ* (∼17 transformants per µg) and the control *myo1*Δ or swe1Δ (∼15 tranformants per µg) insertions of *URA3* on Chr5. C. Southern analysis of *cen5*Δ*::URA3* transformants. Genomic DNA from strains carrying both wild-type *CEN5* homologs (Lanes 1 and 9 (YJB8648)) and *cen5*Δ*::URA3* strains (lanes 2–8, YJB9915, YJB9916, YJB9929, YJB9926, YJB9861, YJB9907 and YJB9909, respectively) were digested with *SnaB*I and *Sac*II, separated by agarose gel electrophoresis and probed with *CEN5* (upper panel) or *URA3* (lower panel) DNA. Position of restriction sites, probe locations and expected sizes of restriction fragments (for both wild-type *CEN5* alleles) is illustrated below. The short *CEN5* homolog was disrupted in strains YJB9915, YJB9861 and YJB9909; the long *CEN5* homolog was disrupted in the other *cen5*Δ*::URA3* strains (YJB9916, YJB9926, YJB9929 and YJB9907). D. Comparative Genome Hybridization (CGH) of Chr5 in *cen5*Δ*::URA3* strains. CGH is displayed as the copy number relative to reference control strain SC5314 using a log2 scale [28],[33]. Black dot, position of *CEN5*. Noncoding sequences flanking *CEN5* were not present on the CGH array. E. CHEF karyotype gel analysis of all *cen5*Δ*::URA3* strains reveals no major alterations in chromosome size in these strains. Lane1 Parental strain RM10 (YJB8648); lane2, YJB9907; lane 3, YJB9909; lane 4, YJB9915; lane 5, YJB9916; lane 6, YJB9926; lane 7, YJB9929 and lane 8, YJB9861. The blot was probed with *URA3* (middle panel) or *CEN5* (right panel). *URA3* replaced *CEN5* in the Chr5 homolog with faster mobility in YJB9907 (lane 2). Chromosome numbers are indicated at left.

We previously identified isochromosome derivatives of Chr5, in which the derived chromosomes replaced either the right arm with left arm information (i(5L)), or replaced the left arm with right arm information (i(5R)) [28],[29]. The only DNA common to the i(5L) and i(5R) isochromosomes is the predicted Chr5 centromere (*CEN5*), including an inverted repeat (*IR*) flanking the central core (*CC5*) (Figure 1A). This *CC5+IR* structure resembles a simplified version of the central portion of typical regional centromeres (e.g., central core+innermost repeats in *S. pombe* centromeres). Telomere truncation constructs [30] replacing either the complete Chr5L arm [29] or the Chr5R arm (Table S1) yielded chromosome fragments (Figure 1A) similar in mitotic stability to that of the isochromosomes (<10^−3^ loss/division). Thus, the *CC5+IR* DNA is associated with centromere function in Chr5 and all its stable derivatives, implying that it provides centromere function.

### Deletion of *CEN5* Results in Stable Chr5 Transformants

To ask if *CEN5* DNA is necessary for centromere function, we replaced *CEN5* (*CC5+IR*) on one of the two Chr5 homologs with a PCR product that included a *URA3* gene flanked by homology to regions just outside the IR (Figure 1B). In *C. albicans* laboratory strains, the two Chr5 homologs differ in at least two ways. First, the mating type-like locus (*MTL*) is heterozygous (*MTLa* or *MTLalpha*). Second, there are two LTR repeats present in the ‘long’ allele of *CEN5* (one in the central core region and one to the left of the left side of the IR) (Figure 1B). In addition, the Major Repeat Sequence (MRS) on Chr5R can vary in size by >50 kb [31],[32].

Initially, we found lower transformation frequencies for *cen5Δ::URA3* constructs than for control *myo1Δ::URA3*, constructs that replaced a gene 100.2 kb from *CEN5*. For example, there were 3–5-fold fewer transformants per microgram of DNA for *cen5Δ::URA3* than for the control when colonies were counted 3–4 days after transformation. The proportion of transformants that carried *bona fide CEN5* deletions, detected by PCR amplification, was also 4–5 fold lower for the *cen5Δ::URA3* than for the *myo1::URA3* strains. However, for the *cen5Δ* transformation (and not for the control transformants), new colonies continued to appear for up to ∼9 days after transformation. After 9 days, similar numbers of Ura^+^ transformants were obtained for the *cen5Δ* and the control transformations (Figure 1B). When the transformant colonies that appeared later were analyzed, the proportion of transformants that carried *bona fide CEN5* deletions, as detected by PCR amplification and confirmed by Southern analysis (Figures 1C and S1A), was similar to, or slightly less than, that of a *myo1Δ::URA3* control strain (Figure 1B). We also obtained transformants in which *CEN5* was replaced with *NAT1*. Southern blots and hybridization to *CEN5* were used to verify the correct insertion of these markers (Figure S1B–D).

**Figure 2:**
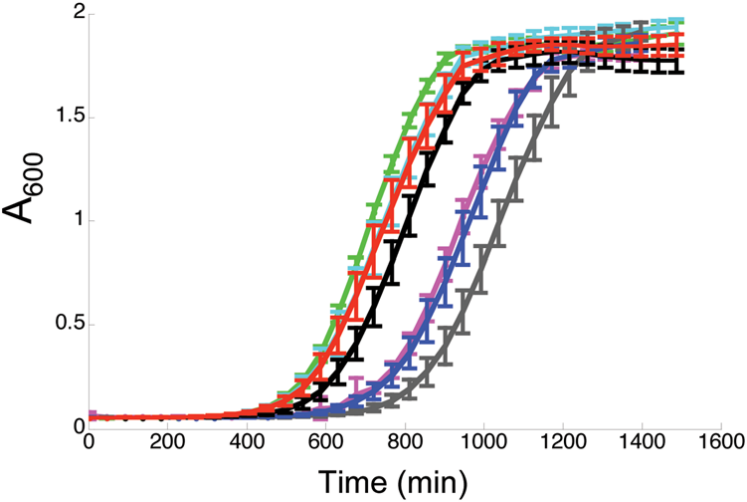
Growth curves for *cen5*Δ*::URA3* strains/*CEN5* strains. Growth rates of wild-type (RM10, black) and *cen5Δ::URA3* strains (YJB9929, green; YJB9909, cyan; YJB9915, red; YJB9907, pink; YJB9926, blue, YJB9916, grey) were measured as described in Materials and Methods. Note that number of cells in the initial culture influences the time when logarithmic division begins and differs between experiments; slope of the logarithmic phase of all curves was not significantly different between experiments (see Table 1). Error bars are shown for every fourth time point to facilitate visualization of the curves.

The growth rates of the *cen5Δ::URA3* strains were indistinguishable from the growth rates of wild-type strains and strains carrying *URA3* at non-centromeric loci (Table 1, Figure 2). Similar levels of stability and growth rates were also seen in the *cen5Δ::NAT1* transformants (Table 1). This result is different from what was seen with *CEN7*: deletion of the Chr7 CENP-A^Cse4p^ binding region was reported to yield only highly unstable chromosomes [23]. Importantly, all of the correct *cen5Δ::URA3* transformants exhibited relatively high levels of mitotic stability (Table 1), measured as the proportion of cells that retain the ability to grow in the absence of uridine.

**Table 1:** Mitotic stability and growth rate of *cen5Δ* strains^1^.

Strain name	Relevant genotype	Mitotic stability (% chromosome retention in selective media)^2^	Loss rate (loss events/ division in non-selective media)	FOA^R^ phenotype^1^	Doubling time (min) (+/−SD)
YJB8648 (RM10)	*ura3::imm434/ura3::imm434 his1::hisG/HIS1*	N/A	N/A	N/A	64.6+/−7.0
YJB9984	*myo1::URA3*	NT	5.65E-7	Loss	59.5+/−5.4
YJB9907	*cen5Δ::URA3/CEN5*	>99.5%	1.72E-5	Loss	64.5+/−5.1
YJB9909	*cen5Δ::URA3/CEN5*	99.5%	1.41E-2	Silencing	64.2+/−5.4
YJB9915	*cen5Δ::URA3/CEN5*	NT	2.06E-2	Silencing	63.4+/−3.7
YJB9916	*cen5Δ::URA3/CEN5*	NT	1.39E-2	Silencing	67.6+/−4.6
YJB9926	*cen5Δ::URA3/CEN5*	NT	1.99E-2	Silencing	62.8+/−5.0
YJB9929	*cen5Δ::URA3/CEN5*	NT	1.49E-7	Loss	56.9+/−6.1
YJB10805	*cen5Δ::NAT1/CEN5*	99.7%	N/A	N/A	74.5+/−6.9
YJB10828	*cen5Δ::NAT1/CEN5*	99.1%	N/A	N/A	68.4+/−6.0
YJB9907-3s	*MTLα/α cen5Δ::URA3/ cen5Δ::URA3*	N/A	N/A	N/A	72.9+/−7.9
YJB9907-6s	*MTLα/α cen5Δ::URA3/ cen5Δ::URA3*	N/A	N/A	N/A	72.1+/−6.8
YJB9929-1s	*MTLα/α cen5Δ::URA3/ cen5Δ::URA3*	N/A	N/A	N/A	66.9+/−5.4
YJB9929-2s	*MTLα/α cen5Δ::URA3/ cen5Δ::URA3*	N/A	N/A	N/A	64.1+/−5.9
YJB9726	*MTLα/α Chr5* homozygous	N/A	N/A	N/A	62.0+/−6.5

1 Silencing indicates colonies appear on SDC-uri after growth on 5-FOA. Loss indicates no re-growth on SDC-uri after selection of 5-FOA.

NT-not tested; N/A—not applicable (strain is either homozygous *URA3/URA3* or is *ura3/ura3*).

Transformation of *C. albicans* sometimes causes unintended aneuploidies [33]. To investigate this possibility, we analyzed the *cen5Δ::URA3* transformants using comparative genome hybridization (CGH) and CHEF karyotype gels. CGH array analysis did not detect any copy number changes in any of the strains: like the parent, all were disomic for all 8 chromosomes (Figure 1D and data not shown). In general, CHEF karyotypes for all but strain YJB9907 were indistinguishable from the parent strain for Chr5 when stained with ethidium bromide (Figure 1E left panel). Only a Chr5-sized band was detected with a *URA3* probe as well as probes for *CEN5*, Chr5L and Chr5R (Figure 1E and data not shown), indicating that no Chr5 rearrangements occurred. In one strain, (YJB9907) the two Chr5 homologs became separable and the two separable Chr7 homologs became inseparable (Figure 1E, left panel, lane 2), most likely because of recombination between the MRS repeats on these chromosomes [34]. The CGH and CHEF gel analysis together indicate that no gross chromosomal rearrangements were evident in the *cen5Δ* strains. Thus, the *cen5Δ* strains retained two intact copies of Chr5 and genome integrity was maintained.

The relative stability of strains lacking *CEN5* implies that a neocentromere, an ectopic assembly of a functional kinetochore onto non-centromeric DNA [35], formed on the Chr5 homologs carrying *cen5Δ* alleles. Importantly, these neocentromeres apparently formed in all recovered transformants in which *CEN5* had been correctly replaced with a selectable marker.

### Cells Lacking Both Copies of *CEN5* Survive and Grow

**Figure 3:**
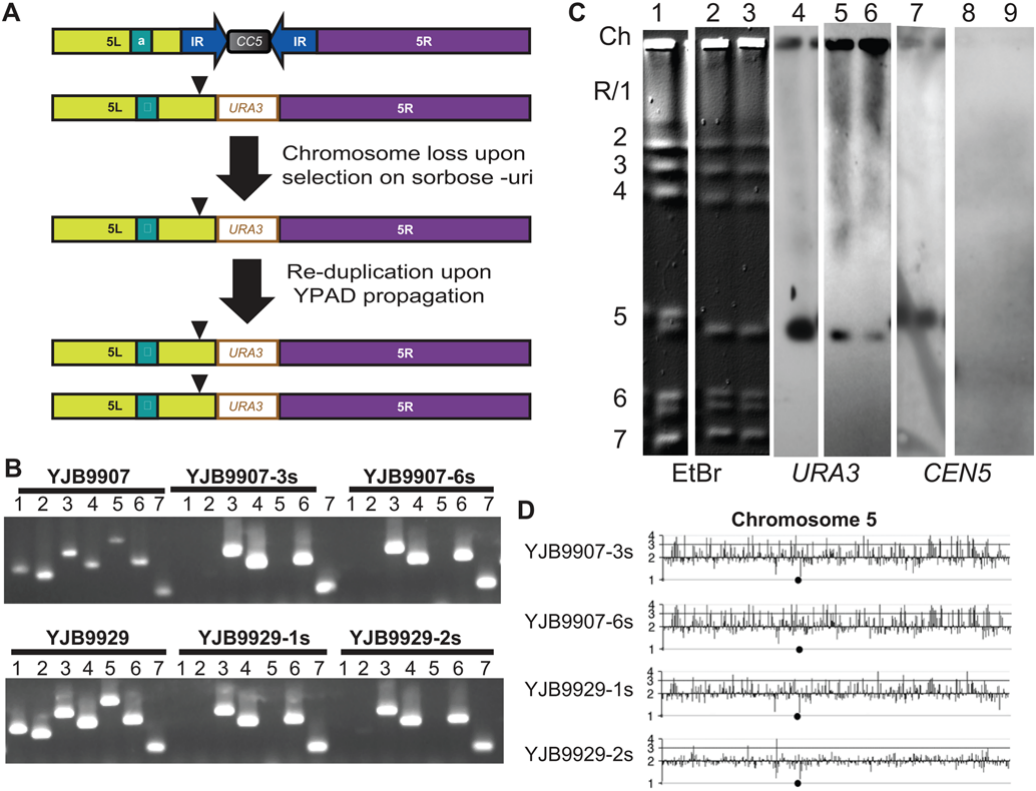
Sorbose selection yields strains with no wild-type *CEN5*. A. Current model for Chr5 homozygosis during and after selection on sorbose selection. B. PCR with centromeric and *MTL* primer pairs (Table S2) reveals heterozygous *Chr5* markers prior to (YJB9907 and YJB9929) and homozygous Chr5 markers following selection on sorbose lacking uridine (YJB9907-3s and -6s, YJB9929-1S and -2S). Lanes 1 and 2, *CEN5* central core region (2898-2901 and 2900-2869); Lanes 3 and 4, *URA3*-*CEN*5 junction (3125-3126 and 725-1915); Lane 5, *MTL* *a* (1193-1194); lane 6, *MTLalpha* (1195-1197); and Lane 7, *CEN4* (2924-2925). C. CHEF karyotype gel of *cen5Δ* strains following sorbose treatment. Lanes 1–3, ethidium bromide staining of strains YJB9907, YJB9907-3s and YJB9907-6s, respectively. Lanes 4–6 and 7–9 are Southern blots of the same gel lanes probed with *URA3* or *CEN5* sequences. Similar results were obtained for YJB9929, YJB9929-1s and YJB9929-2s (data not shown). Chromosome numbers at left are as in Figure 1E. All strains were run on the same gel as Figure 1E and YJB9907 data is repeated here to emphasize the relative band mobilities. D. Comparative Genome Hybridization (CGH) for Chr5 in *cen5Δ::URA3* post-sorbose strains. CGH is displayed as in Figure 1D. All *cen5Δ::URA3* sorbose derivative strains are disomic for Chr5, indicating a second non-disjunction event resulting in a second copy of Chr5 after loss of the WT allele on sorbose.

Because *C. albicans* is diploid, all of the *cen5Δ::URA3* strains retained one wild-type copy of *CEN5*. While centromeres generally function only *in cis*, phenomena that act in *trans*, such as transvection in Drosophila (reviewed in [36]), raised the possibility that the *cen5Δ::URA3* homolog was able to segregate through some interaction with the wild-type *CEN5* homolog of Chr5. To determine if cells could survive and divide in the absence of any *CEN5* DNA, we utilized sorbose growth, which selects for cells that lose one copy of Chr5 [37]. Strains were plated to sorbose medium lacking uridine to select for Ura^+^ cells carrying only one Chr5 homolog (Figure 3A, middle panel). The colonies that appeared on sorbose-uri medium were then streaked to rich medium (YPAD) and larger colonies (those that likely reduplicated the single copy of Chr5 [37], Figure 3A, lower panel) were streaked to SDC-uri to ensure that they retained the *URA3* marker. PCR analysis confirmed retention of the *cen5Δ::URA3* and failed to detect wild-type *CEN5* or *MTLa* (Figure 3B) in several isolates, consistent with the idea that the wild-type Chr5/*CEN5* homolog had been lost. CHEF analysis indicated that the karyotype of these strains was unaltered (Figure 3C). In the case of YJB9907, the parental Chr5 homologs had distinct mobilities, and the sorbose-selected derivatives (YJB9907-3s and YJB9907-6s) carried only the smaller homolog, which hybridized to *URA3* and not to *CEN5* (Figure 3C, lanes 5 and 8). CGH analysis indicated that the remaining Chr5 homolog was present in two copies (Figure 3D) following growth on SDC-uri. For YJB9929, the two derivatives (9929-1s and 9929-2s) showed similar results to YJB9907 and its derivatives. Both PCR and Southern analysis confirmed that the original *CEN5* DNA was absent from both strains (Figure 3B&C).

The four strains lacking any *CEN5* DNA exhibited growth rates that were slightly slower than the growth rates of wild-type and *cen5Δ::URA3/CEN5* strains (Figure S1E, Table 1). This reduced fitness could be because the cells lacked *CEN5* DNA completely or it could be due to the homozygosis of all markers on Chr5 that occurred as a consequence of sorbose selection and subsequent growth on rich medium (Figure 3A). To address this question, we performed growth rate experiments with strain YJB9726, which maintained and then reduplicated one *CEN5* homolog, yet was homozygous for all genes on Chr5 because it had been propagated on sorbose (Table 1, Figure S1E). Importantly, the growth rates of strains with and without intact *CEN5* were similar, indicating that *CEN5* DNA is not necessary for chromosome propagation and stable strain maintenance. Rather, the slower growth is likely due to one or more genes that, when homozygous, cause reduced fitness. This supports the idea that neocentromeres formed in the *cen5Δ::URA3* strains have no obvious deleterious effect on growth rate or fitness in standard laboratory growth conditions.

### Two Classes of Neocentromeres in *cen5Δ::URA3* Transformants Differ in *URA3* Silencing

**Figure 4:**
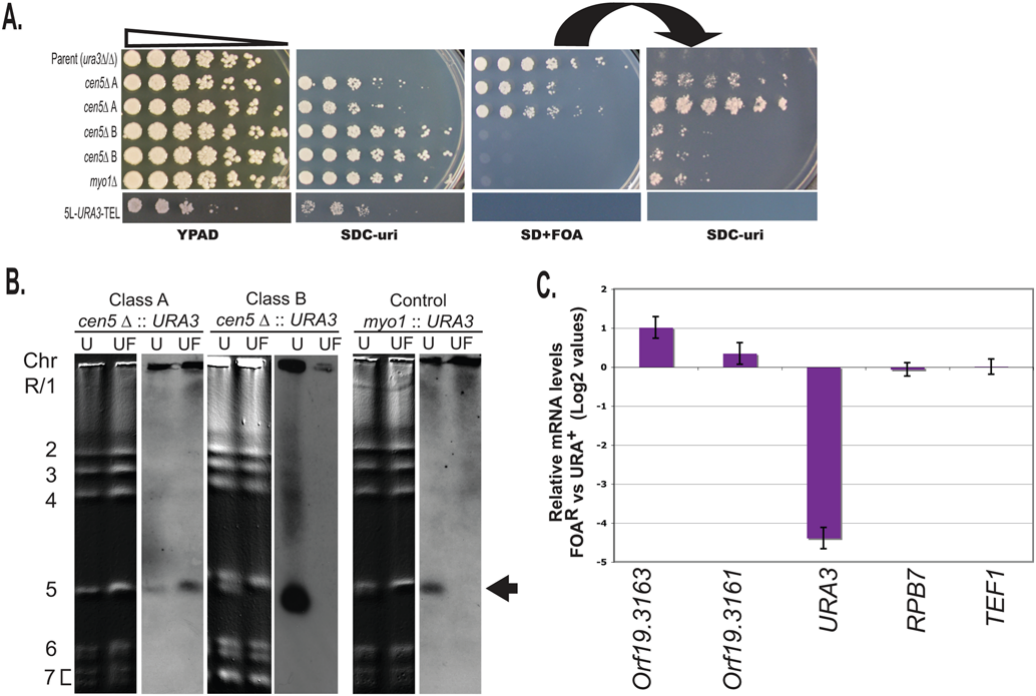
Class A *cen5*Δ*::URA3* strains undergo reversible transcriptional silencing. A. Reversible silencing of Class A *cen5Δ::URA3* transformants. Five-fold serial dilutions of cultures were plated onto rich medium (YPAD), medium selective for *URA3* expression (SDC-uri) and medium selective for Ura^−^ cells (SD+FOA). Cells on the SD+FOA plate were then replica plated to SDC-uri (right panel). Strains from top to bottom: YJB8648 (parent), YJB9909 and YJB9916 (*cen5Δ::URA3* Class A, short and *CEN5* long allele deleted, respectively), YJB9861 and YJB9907 (*cen5Δ::URA3* Class B, long and short *CEN5* deleted, respectively) and YJB9984 (*myo1Δ::URA3*) and YJB9779 (5L-*URA3*-TEL). B. CHEF Southern analysis of Ura^+^ (U) transformant and derivatives that became FOA^R^ (UF). Class A (YJB9909) and Class B (YJB9907) and control strains (YJB9984) grown in SDC-uri (U) or transferred from SDC-uri to SD+FOA and propagated in SD+FOA (UF), as indicated. Left panels (Lanes 1, 2, 5, 6, 9, and 10), DNA stained with ethidium bromide; right panels, Southern analysis with *URA3* probe. *URA3* is not detected in FOA^R^ Class B and *myo1Δ::URA3* strains, but is retained in the FOA^R^ Class A strain (arrow). In strain YJB9907 (Class B), *URA3* inserted into the faster migrating *CEN5* homolog (see Figures 1E and 3C). C. Transcriptional silencing of *URA3* in Class A transformants grown on 5-FOA. cDNA from Class A strains YJB9909 and YJB9916 was analyzed by quantitative real time-PCR (qRT-PCR) and the average log2 ratio of expression in each strain grown in SD+FOA was measured relative to expression of the same strain grown in SDC-uri. The average log2 ratio of the two strains is displayed. Control genes *RPB7* and *TEF1* exhibited no significant change in expression between the two growth conditions. Orf19.3161 and Orf19.3163, located ∼1.5 and 2.5 kb, respectively, to the left of *CEN5* (Figure 7A), are expressed at slightly higher levels when grown in SD+FOA relative to SDC-uri. Error bars represent the standard error of the mean for each gene.

Two different classes of *cen5Δ::URA3* transformants were distinguishable based on the rate at which cells acquire the ability to grow on 5-FOA (FOA^R^ cells), a compound that is toxic to Ura^+^ cells [38]. This assay is generally used as a proxy for mitotic loss rates and quantified using fluctuation analysis to identify median FOA^R^ rates [39],[40]. The Class A transformants, termed ‘proximal neoCENs’ based on data described below, became resistant to 5-FOA at a rate of ∼10^−2^–10^−3^/cell division and the phenotype was reversible. The Class B transformants (termed ‘distal neoCENs’ based on data described below) became irreversibly resistant to 5-FOA at a rate of ∼10^−5^–10^−6^/cell division, similar to the rate of 5-FOA resistance of *myo1Δ::URA3* strains (Figure 4A). Disruption of either Chr5 homolog (short or long, Figure 1B) yielded both Class A and Class B transformants, indicating that the class of transformant was not due to disruption of a specific *CEN5* allele.

**Figure 5:**
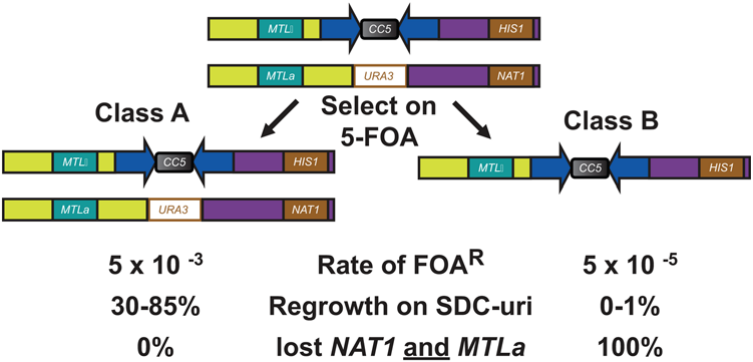
Genetic segregation of markers in *cen5*Δ*::URA3* transformants. Model for marker segregation if *URA3* is silenced (left) or Chr5 is lost (right). In parental strain YJB10064 the two homologs of Chr5 are distinguishable because one copy of *HIS1* (on Chr5R) was replaced with *NAT1* and *MTL* (on Chr5L) is heterozygous. YJB10064 was transformed to replace *CEN5* with *URA3* and transformants with higher (∼10^−3^, Class A) and lower (∼10^−5^, Class B) FOA^R^ rates were identified. Class A FOA^R^ strains retained both the *NAT1* and *HIS1* markers. Class B FOA^R^ strains lost both *NAT1* and *MTLa*, consistent with loss of the Chr5 homolog carrying *cen5Δ::URA3*. Similarly, both markers were lost in FOA^R^ derivatives of *the myo1::URA3* control strain (YJB10169).

Southern analysis of the Class B FOA^R^ strains indicated that, like *myo1Δ::URA3* transformants, when these cells became FOA^R^, they had lost the *URA3* marker, (Figure 4B). Thus, rare FOA^R^ derivatives of Class B *cen5Δ::URA3* transformants have either undergone a non-disjunction event to lose the entire chromosome carrying *cen5Δ::URA3 or* have lost the *URA3* via recombination. To distinguish between these possibilities, we followed the segregation of markers on both arms of Chr5 (*HIS1/his1Δ::NAT1* near the Chr5R telomere and *MTL* on Chr5L). At least 98% of the FOA^R^ Class B derivatives had lost heterozygosity at *HIS1* (on Chr5R) and, among those, 100% of them also lost heterozygosity at *MTL* (on Chr5L) (Figure 5). Thus, there is strong genetic support for the idea that whole chromosome loss was the cause of the FOA^R^ phenotype in Class B transformants (Figure 5).

Several lines of evidence indicated that the higher rate of FOA^R^ in Class A transformants was due to transcriptional silencing and not loss of *URA3* DNA. First, the FOA^R^ phenotype was reversible: transformants initially selected on SDC-uri gave rise to cells that subsequently grew on SD+5-FOA and these colonies could subsequently give rise to cells that grew on SDC-uri (Figure 4A). In each transfer (from SDC-uri (U) to SD+FOA (UF), then to SDC-uri (UFU), then to SD+FOA (UFUF) and so on), only a subpopulation of colonies appeared (∼1/1000 cells). Importantly, the reversible FOA^R^ phenotype was unique to the *cen5Δ::URA3 strains*; when *URA3* was inserted adjacent to an intact *CEN5* (e.g., in the 5L-*URA3*-TEL strain), it was not reversibly silenced (Figure 4A). Second, strains grown on SD+FOA retained *URA3* DNA on Chr5, as detected by CHEF Southern (Figure 4B) and PCR (data not shown). Third, *URA3* DNA sequence was unaltered in any of the FOA^R^ Class A transformants (YJB9909, YJB9915, YJB9916 and YJB9926, data not shown), indicating *URA3* did not acquire point mutations. Fourth, genetic analysis of Class A FOA^R^ transformants carrying markers on Chr5L and Chr5R did not lose markers from either arm (Figure 5), reinforcing the idea that both Chr5 homologs were retained on 5-FOA. Finally, real time PCR detected ∼16 fold lower levels of *URA3* mRNA in cells grown on SD+FOA relative to those grown on SDC-uri. This indicates that *URA3* expression was silenced at the transcriptional level in the Class A FOA^R^ cells (Figure 4C). Taken together, these results indicate that *URA3* DNA was silenced at the transcriptional level in most Class A FOA^R^ transformants and therefore, that the *cen5Δ::URA3* Chr5 homolog was stable to a similar degree in these cells and in the Class B cells. Thus, *cen5Δ* chromosomes are very stable, far in excess of the stability of minichromosomes in either *S. cerevisiae* or *S. pombe* that bear *bona fide* centromeres [41],[42]. This provides more evidence that the *cen5Δ::URA3* homologs in both classes of transformants must carry a functional neocentromere.

### Class A Neocentromeres Are Located Proximal to *cen5Δ::URA3*

Active regional centromeres and neocentromeres are, by definition, associated with CENP-A^Cse4p^ [10]–[12],[43],[44]. To identify the position of neocentromeres on *cen5Δ::URA3* homologs, we performed chromatin immunoprecipitation (ChIP) experiments using antibody raised against an N-terminal peptide from *C. albicans* CENP-A^Cse4p^ (see Materials and Methods). We first analyzed Class A transformants to investigate the mechanisms linking neocentromere function and the growth phenotypes on 5-FOA or medium lacking uridine in these strains.

**Figure 6:**
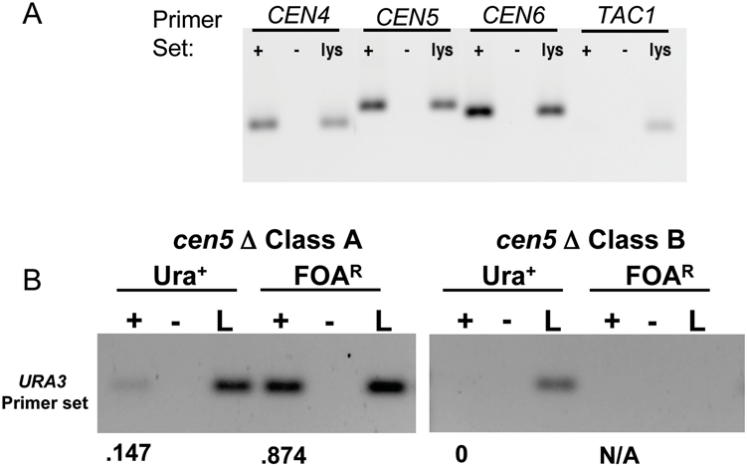
Chromatin immunoprecipitation (ChIP) of CENP-A^Cse4p^ detects centromere DNA. A. CENP-A^Cse4p^ antibody specifically recognizes *C. albicans* centromeric DNA. Chromatin immunoprecipitation (ChIP) of wild-type strain SC5314. PCR amplification of *CEN4*, *CEN5*, *CEN6* or non-centromeric *TAC1* DNA from wild-type strain SC5314. Lysates (lys) were precipitated with (+) or without (−) CENP-A^Cse4p^ antibody. B. CENP-A^Cse4p^ antibody precipitates more *URA3* in FOA^R^ cells than in Ura^+^ cells. PCR amplification after chromatin immunoprecipitation (ChIP) was performed for Class A (YJB9907) and Class B (YJB9929) *cen5Δ::URA3* strains using primers that amplify the *URA3* gene that replaced the *CEN5* DNA sequences. In Class B strains, *URA3* did not amplify from the FOA^R^ lysate because the *cen5Δ::URA3* Chr5 homolog had been lost (Figure 4B). Numbers indicate the intensity of the +antibody (+) band relative to the lysate band for each sample.

As expected, anti-CENP-A^Cse4p^ specifically recognized DNA in *CEN4*, *CEN5* and *CEN6* central core regions, but did not preferentially precipitate non-centromeric sequences such as the *TAC1* gene on Chr5L (Figure 6A). In addition, in Class A transformants grown on SD+5-FOA, CENP-A^Cse4p^ preferentially associated with *URA3*, while when these strains were grown on SDC-uri, CENP-A^Cse4p^ associated with *URA3* to a lesser degree (Figure 6B). This implies that the reversible silencing is due to reversible association of CENP-A^Cse4p^ with *URA3*. In contrast, in Class B Ura^+^ cells, CENP-A^Cse4p^ was not associated with *URA3*, and in FOA^R^ cells the *URA3* DNA had been lost from the strain and was not detectable in the lysate (Figure 6B).

**Figure 7:**
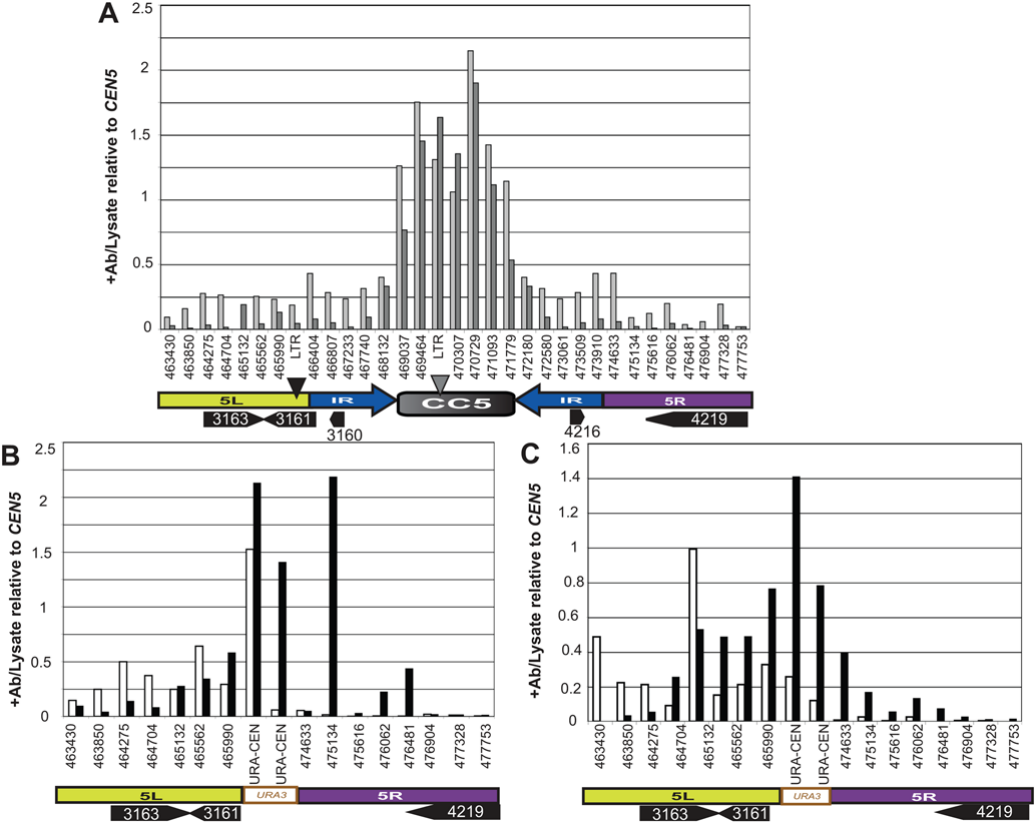
Chromatin Immunoprecipitation detects the position of wild type and Class A (proximal) neocentromeres. A. Contiguous ChIP PCR of *CEN5* in wild-type strains SC5314 (wt1, light gray) and RM10 (wt2, dark gray). Contiguous PCRs were performed for ∼15 kb of sequence flanking and including *CEN5* with primer pairs amplifying ∼400 bp each of DNA across this region (see schematic below the graph (Ch5L (yellow), Ch5R (purple), *CC5* (*CEN5* central core (gray)) and flanking inverted repeat (blue)). Predicted ORFs in the region are illustrated with black arrows. Results are expressed as the ratio of signal for CENP-A^Cse4p^ antibody-specific PCR products relative to the abundance of the product in the untreated lysate. For each strain a representative ChIP sample of at least two biological replicates is displayed. In each extract, only *CC5* DNA was significantly associated with CENP-A^Cse4p^. Correlation analysis indicated that the two wild-type strains were similar (correlation coefficient = 0.80). B. Contiguous ChIP PCR of region including and flanking the *cen5Δ::URA3* insertion in Class A strain YJB9909 for cells grown in SDC-uri (white) or selected on 5-FOA (black). Data displayed represents a representative ChIP sample of 3 separate biological replicate extracts for each growth condition. Correlation analysis [70] indicated that the two growth conditions did not correlate well (correlation coefficient = 0.40). C. ChIP of Class A strain (YJB9909) after three rounds of alternating selection on SDC-uri and SD+FOA. Contiguous ChIP PCR of *cen5Δ::URA3* homolog and flanking chromosome arms grown in SDC-uri (white bars) and SD+FOA (black bars). The results from the two different growth conditions are not well correlated (correlation coefficient = 0.47).

To more precisely localize the active neocentromeres in Class A transformants, we analyzed DNA from anti-CENP-A^Cse4p^ ChIP experiments using PCR amplification of contiguous 400 bp DNA segments spanning a ∼15 kb region including and surrounding *CEN5*. Consistent with previous studies [22],[23], CENP-A^Cse4p^ was associated with the *CC5* sequence and not with the IR or flanking Ch5L or Ch5R sequences (Figure 7A) in two different parental strains that each carried intact copies of both *CEN5* homologs. Furthermore, in six independent *cen5Δ::URA3* transformants, the wild-type *CC5* DNA on the intact, unmodified homolog (which could be distinguished based on the presence or absence of the LTR insertion, Figure 1B) was specifically associated with CENP-A^Cse4p^ (Figure S2A and B). Thus, in all *cen5Δ::URA3* strains, the centromere on the wild-type Chr5 homolog was not perturbed by the loss of *CEN5* on the other homolog.

In addition, CENP-A^Cse4p^ was associated with DNA ∼1 kb to the left of *URA3* in all Class A transformants growing on SDC-uri. A similar pattern was seen in four independent transformants, including those carrying deletions of either *CEN5* homolog (with and without the LTR) (Figures 7B and S2C). In contrast, when Class A transformants were grown on 5-FOA (*URA3* gene silenced), CENP-A^Cse4p^ always associated with *URA3* in all four independent Class A transformants, irrespective of the *CEN5* homolog that had been disrupted (Figures 7B, S2C and data not shown). Importantly, when strains were cycled from SDC-uri to 5-FOA and then back to SDC-uri for two additional cycles (UFUFU) and then to SDC+FOA again (UFUFUF), the same patterns were retained: in cells grown on 5-FOA the CENP-A^Cse4p^ was associated mostly with *URA3*; in cells grown on SDC-Uri CENP-A^Cse4p^ enrichment was adjacent to the *URA3* gene (Figure 7C). This suggests that the neocentromere occupied different positions when Class A cells were grown under different conditions and that its average position had shifted position by ∼1–2 kb.

Analysis of Class A transformants in which the short vs long *CEN5* homologs were disrupted revealed that the *episemon* LTR was only associated with CENP-A^Cse4p^ when the LTR was linked to the *cen5Δ::URA3* homolog (Figure S2C). In addition, the LTR appeared to be a preferred site for CENP-A^Cse4p^ binding. When it was located on the same homolog as *cen5Δ::URA3*, the lateral spread of CENP-A^Cse4p^ from *URA3* into the adjacent DNA was reduced. Thus, CENP-A^Cse4p^ associated with DNA flanking the left side of the IR only *in cis* and not *in trans*.

In the proximal neoCEN transformants, we detected the association of CENP-A^Cse4p^ with *URA3* because of the FOA^R^ counter-selectable phenotype of Ura^−^ cells. Quantitative RT PCR indicated that the 16-fold silencing of *URA3* occurred by affecting levels of mRNA (Figure 4C). To ask if association with CENP-A^Cse4p^ resulted in transcriptional silencing of genes other than *URA3*, we measured the expression levels of two genes located just left of *cen5Δ::URA3* in Class A/proximal neoCEN strains. These two genes (orf19.3161 and orf19.3163) were expressed at higher levels in FOA^R^ cells (when CENP-A^Cse4p^ was not associated with them) relative to their expression in Ura^+^ cells (when CENP-A^Cse4p^ was associated with them) (Figure 4C). This is consistent with the idea that association of a gene with CENP-A^Cse4p^ results in decreased transcription from that gene.

### Class B Neocentromeres Form at Loci Distal to *cen5Δ::URA3*

In the Class B strains, CENP-A^Cse4p^ associated with *CC5* on the wild-type homolog and was not associated with other DNA fragments near *cen5Δ::URA3* (Figure S2D). This suggested that the neocentromeres in Class B strains were located beyond the 15 kb region analyzed by ChIP, and thus are located too far from *cen5Δ::URA3* to silence it. To locate the position(s) of neocentromeres in Class B transformants, we performed ChIP followed by massively parallel high-throughput sequencing (ChIP-SEQ) of one wild-type and two Class B transformants (YJB9907 and YJB9929). As expected, in each strain, the major region associated with CENP-A^Cse4p^ on each wild-type chromosome overlapped with the region previously described by Carbon and co-workers as the centromere [22],[23], although it was smaller, ranging from 2–3.3 kb (Figure S3). The difference in sizes predicted is likely due to the improved resolution from the smaller average fragment size used in the ChIP pulldowns.

**Figure 8:**
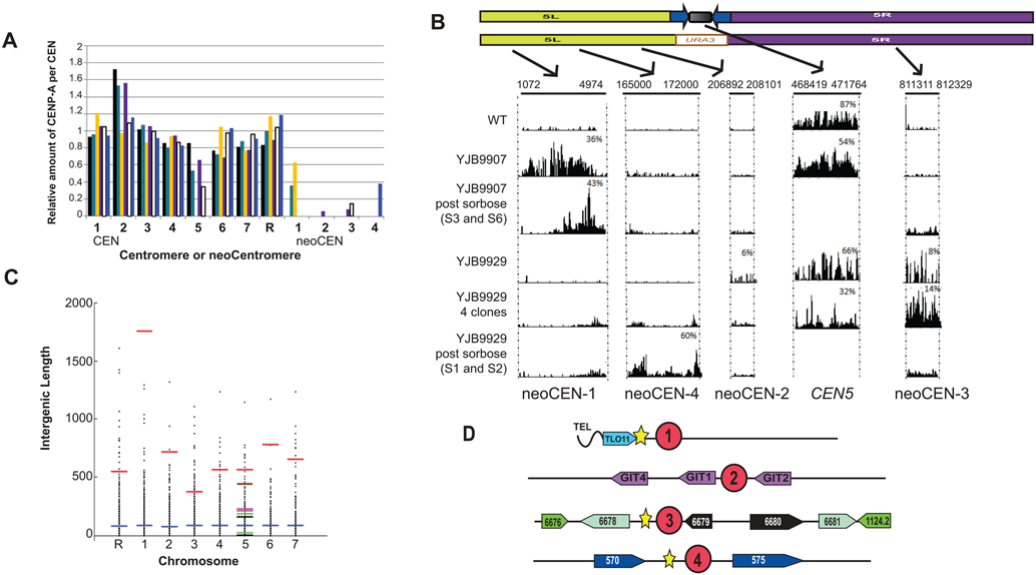
ChIP-SEQ detects position of *CEN5* Class B (distal) neocentromeres. A. Relative number of sequence reads for each wild-type centromere and neocentromere in parent strain YJB8648 (black), *cen5Δ::URA3* strains YJB9907 (teal) and sorbose-selected derivativesYJB9907 3s and 6s (yellow), YJB9929 (purple), four independent YJB9929 colonies (white) and sorbose-selected derivatives YJB9929-1s and 2s (blue). NeoCEN-1 is detected only in YJB9907 and YJB9907s, neoCEN-2 was detected only in the original YJB9929 isolate while neoCEN-3 appeared in the original YJB9929 isolate, as well as independent colonies derived from it. NeoCEN-4 appeared in YJB9929s and was not detected in other isolates. Number of reads was normalized to the average number of reads per centromere for *CENs R*, *1*, *2*, *3*, *4*, *6 and 7*. The number of *CEN5* reads decreased in strains with only one copy of *CEN5* and was absent in strains lacking *CEN5*. The number of reads on *CEN2* is 50% higher than for other *CENs* in YJB8648, YJB9907 and YJB9929 because Chr2 is trisomic in these strains. B. ChIP-SEQ reads on chromosome 5 for strain YJB9907 and YJB9929 and its derivatives. Chromosome coordinates of peaks are shown below the Chr5 diagram for the *CEN5* and the 4 neoCEN regions identified. Histograms display number of sequence reads for the region in each strain. For YJB9929 colonies, ChIP extracts of four independent colonies were pooled for sequence analysis. YJB9907 post-sorbose and YJB9929 post-sorbose include sequencing data for two independent colonies that were pooled after ChIP. Number of sequence reads relative to the average number of reads for wild-type centromeres in that strain are expressed as percentages. C. Sizes of intergenic regions for centromeres and neocentromeres. All intergenic regions (black dots) are shown for each chromosome (X-axis). Bars indicate average intergenic region size for each chromosome (blue) and wild-type centromere (red) on each chromosome. Sizes of intergenic regions flanking ORFs within neoCEN-1 (black), neoCEN-2 (red), neoCEN-3 (green) and neoCEN-4 (brown) are indicated. D. Organization of neocentromere regions. Position of neoCENs is indicated by the numbers in the red circles. LTR repeats are noted as yellow stars. ORFs (colored arrows) flanking the neocentromeres are indicated with their gene name or orf19 number. ORFs with high levels of identity have the same color. *TLO11* is similar to *TLO* genes at all other telomeres. Two ORFs to the left of neoCEN-3 are highly similar to two ORFs to the right of the neocentromere.

In all three strains, the degree to which each centromere, other than *CEN5*, associated with CENP-A^Cse4p^ was similar relative to the other centromeres (Figure 8A). In both of the *cen5Δ::URA3/CEN5* strains, CENP-A^Cse4p^ associated with the remaining *CEN5* copy at a level slightly higher than half the relative signal present on two copies of *CEN5* in wild-type cells (Figure 8A).

Analysis of the ChIP-SEQ data also identified new CENP-A^Cse4p^-associated DNA sequences on Chr5 in both Class B neocentromere strains (Figure 8B). In YJB9907 CENP-A^Cse4p^ was associated with a ∼4 kb region near the left telomere. This peak (present in one copy, termed neoCEN-1) included 36% of the ChIP-SEQ reads relative to the average centromere (present in two copies). This is ∼half as much CENP-A^Cse4p^ as was present in the *CEN5* peak from the other Chr5 homolog. In strain YJB9929, two different regions of the genome exhibited small peaks of CENP-A^Cse4p^ association: ∼1.2 kb around orf19.1978 on Chr5L (termed neoCEN-2) and a ∼1 kb region overlapping orf19.6678 and it's 5′ upstream region on Chr5R (termed neoCEN-3) (6% and 8% of the signal seen in an average centromere, respectively). This suggests that the neoCENs associate with less CENP-A^Cse4p^ than does a wild-type centromere.

The positions of the predicted neocentromeres in the two Class B strains, as well as in wild-type strains, were then analyzed by standard ChIP analysis, using primers that amplified fragments across the regions identified by ChIP-SEQ (Figure S4). As predicted from the ChIP-SEQ analysis, in YJB9907 CENP-A^Cse4p^ was associated with unique, telomere-adjacent sequence within and 5′ to orf19.5698 (neoCEN-1), and in YJB9929 CENP-A^Cse4p^ was associated with neoCEN-2 and neoCEN-3 DNA. Thus, neocentromeres can form at positions far from the deleted *cen5Δ::URA3* locus. For this reason, we term Class B transformants ‘distal neoCEN’ transformants as compared to the Class A ‘proximal neoCEN’ transformants.

Importantly, the neocentromere peaks were strain-specific. They never appeared in the wild-type strain or in the other neocentromere strain (Figure 8B). ChIP extracts of YJB9907 analyzed by PCR confirmed the presence of CENP-A^Cse4p^ near the left telomere and its absence in the neoCEN-2 or neoCEN-3 regions. Similarly, in YJB9929, CENP-A^Cse4p^ was associated with neoCEN-2 and neoCEN-3 and not with the neoCEN-1 region. Furthermore, on all other chromosomes, telomeric regions were not associated with CENP-A^Cse4p^ (data not shown). Thus, one unambiguous neocentromere is formed in YJB9907, while in YJB9929 at least two different DNA regions are associated with CENP-A^Cse4p^ and presumably function as neocentromeres.

Because dicentric chromosomes are highly unstable [45],[46] and because YJB9929 exhibited a growth rate similar to that of YJB9907 and wild-type strains (Table 1, Figure 2), we hypothesized that the YJB9929 ChIP extract was prepared from a mixed population of cells carrying at least two different neocentromeres. To test this possibility, we analyzed 4 independent colonies from the YJB9929 culture by ChIP-SEQ. Despite the presence of a mixed population, we detect CENP-A^Cse4p^ peaks only at the neoCEN-3 position (as well as at the original *CEN5* position, which is expected because this strain is heterozygous for *CEN5/cen5Δ::URA3* (Figure 8B)). PCR of these ChIP extracts confirmed that CENP-A^Cse4p^ is located at the neoCEN-3 position in 3 of the 4 colonies (Figure S4). This is consistent with the stronger signal for neoCEN-3 relative to neoCEN-2 in the original YJB9929 isolate (Figure 8B). Thus, in different strains, neocentromeres formed at different loci and on both arms of Chr5.

### Distal Neocentromere Position Can Move upon Sorbose Selection

We next determined the position of the neocentromeres in the sorbose-derived YJB9907s and YJB9929s strains lacking both wild-type *CEN5* homologs. CENP-A^Cse4p^ ChIP-SEQ of strains YJB9907-3s and YJB9907-6s detected CENP-A^Cse4p^ associated with neoCEN-1, near the Chr5L telomere (Figure 8B), although the signal peak appeared to shift to the telomere-distal portion of the peak. PCR analysis of the two strains confirmed that CENP-A^Cse4p^ remained associated with this telomere-adjacent region. Furthermore, no major peak was detected at any other Chr5 locus tested (e.g, neoCEN-2 or neoCEN-3, Figure S4) and, consistent with the loss of *CEN5* in these strains, no *CEN5* DNA was associated with CENP-A^Cse4p^. Thus, despite the fact that sorbose growth causes stresses that can result in chromosome loss, the neocentromere in strain YJB9907 was sufficiently stable to be maintained in the same general region during selection for Chr5 loss and reduplication/non-disjunction. Nonetheless, the shift in the peak position suggests that the neoCEN in YJB9907s strains may have undergone a local shift in its position.

In contrast, ChIP of YJB9929-1s and YJB9929-2s did not detect CENP-A^Cse4p^ associated with neoCEN-1, neoCEN-2 or neoCEN-3 (Figure S4). ChIP-SEQ of the two YJB9929-s strains identified a new, strong CENP-A^Cse4p^ signal associated with sequences ∼170 kb from the Chr5L telomere (Figure 8B, neoCEN-4). PCR of ChIP samples from the two independent derivatives (Figure S4) confirmed that CENP-A^Cse4p^ was present at this locus. Thus, in strain YJB9929 exposed to the stress of growth in sorbose, the original neoCEN locations were lost and a new active neocentromere (neoCEN-4) arose at a distance of ∼640 kb from the neoCEN-3.

### Properties of DNA at Neocentromeres

It has been suggested that analysis of neocentromere DNA will identify features of DNA sequence necessary for centromere function [11]. The only characteristic common to all *C. albicans* centromeres is that they are found in very long intergenic regions (average 7.5 kb, range 3.8–17.4 kb) (Figure 8C and [23]). In addition, seven of the eight *C. albicans* centromeres are flanked by repeats; four (including *CEN5*) are oriented as inverted repeats while three are oriented as direct repeats.

Like other regional centromeres, *C. albicans* centromeres have no common primary DNA sequence feature [23]. Consistent with this, no sequence motifs common to centromeres and/or neocentromere regions (including flanking sequences upstream and downstream of the centromeres and neocentromeres) were identified using stringent search criteria and no obvious inverted repeats, palindromes or common sequence motifs were found at all centromeres and neocentromeres (using EMBOSS (http://pro.genomics.purdue.edu/emboss/) [47], JSTRING (http://bioinf.dms.med.uniroma1.it/JSTRING) and MEME [48]). Furthermore, neither the centromeres nor neocentromeres were enriched for any nucleotide or dinucleotide combination relative to total genomic DNA.

Importantly, the *C. albicans* neocentromeres identified here all shared two features seen at *bona fide* centromeres. First, for each of the neocentromeres, at least one intergenic region within the CENP-A binding peak was considerably larger than the average size of intergenic regions on Chr5 (902 bp) or across the entire genome (853 bp) (Figure 8C). Furthermore, all of the neocentromeres were found in close proximity to repeat sequences (Figure 8D). NeoCEN-1 is immediately next to the telomere-adjacent repeated *TLO11* and the rho-5a LTR repeat and within 4 kb of the telomere repeats. NeoCEN-2 is centered on *GIT2*, with adjacent copies of *GIT1* and *GIT4*, which encode predicted glycerophosphoinositol permeases that share 75% and 73% identity with *GIT2* at the DNA level (determined by BLAST [49]). NeoCEN-3 is flanked by an inverted repeat that includes orf19.6676 and orf19.6678 on the left and orf19.6681 and orf19.1124.2 on the right. These gene pairs are 99% (orf19.6676 and orf19.1124.2) and 70% (orf19.6678 and orf196681) identical. In addition, the intergenic region adjacent to orf19.6678 includes an omicron-5a LTR element. Finally, neoCEN-4 is within a large (>4.4 kb) intergenic region that includes an LTR element (chi-5a) and is flanked by orf19.570 and orf19.575, which are >75% identical at the DNA sequence level and are organized in a direct repeat orientation. Thus, all four neoCENs include a large intergenic region as well as inverted or direct repeats. Furthermore, three of the four neoCENs also feature an LTR repeat in close proximity.

## Discussion

This study has revealed important new properties of *C. albicans* small regional centromeres, many of which are reminiscent of the properties of larger regional centromeres. First, the loss of centromere DNA results in formation of a stable neocentromere. *CEN5* can be replaced with *URA3* or *NAT1* and, in *bona fide* transformants, the resulting Chr5 remains stable and cells are viable in the absence of any *CEN5* DNA. Second, neocentromere formation is highly efficient, apparently occurring in all *bona fide* transformants. Third, while *C. albicans* lacks classic heterochromatin components such as HP1, histone H3K9 methyltransferases or DNA methyltransferases, an active centromere silences transcription of an associated gene. Fourth, neocentromeres can form at several different regions of Chr5, including within each of the chromosome arms and at a telomere-adjacent position. Fifth, loci that form neocentromeres have two common properties: a longer-than-average intergenic region and the presence of repeated DNA. Finally, under selective conditions, neocentromeres can move either locally (a few kb) or over long distances (100's of kb).

**Figure 9:**
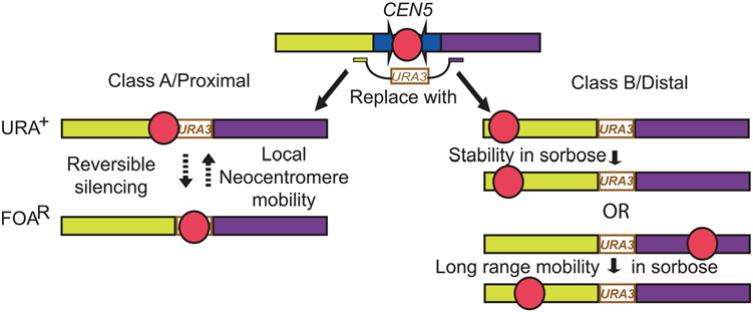
Model for neocentromere formation in *C. albicans*. On wild-type Chr5, CENP-A^Cse4p^ and other kinetochore proteins (red) bind only within the *CC5* region. Replacement of *CEN5* with *cen5Δ::URA3* and selection for *URA3* expression in the transformants results in the formation of Proximal (Class A) and Distal (Class B) neocentromeres. Proximal neocentromere formation initially allows expression of *URA3*; growth in SD+FOA selects for cells in which CENP-A^Cse4p^ has silenced *URA3* by associating with it. This silencing and movement of the neocentromere over several kb is reversible. Distal neocentromeres form at several potential sites on either arm of Chr5 and do not interfere with *URA3* expression. Upon sorbose selection for Chr5 homozygosis, they can be relatively stable (as in YJB9907) or can move to a new position.

Taken together, our data suggest a model (Figure 9) in which all recovered transformants form a neocentromere, defined by the presence of CENP-A^Cse4p^. In some transformants, the neocentromere assembles adjacent to *cen5Δ::URA3* and subsequent selection for FOA^R^ identifies those individuals in which *URA3* is silenced by virtue of its association with CENP-A^Cse4p^. Because this silencing and the association of CENP-A^Cse4p^ with *URA3* are reversible, we propose that direct association with CENP-A^Cse4p^ nucleosomes is incompatible with active transcription and that the neocentromere moved onto the *URA3* gene and silenced it in FOA^R^ cells. In other transformants, the neocentromere assembles far from the deleted centromere and FOA^R^ derivatives are usually due to chromosome loss. Furthermore, while exposure to sorbose promotes Chr5 loss in *C. albicans* [37], here we found that the telocentric neoCEN-1 remained relatively stable upon sorbose growth while, in a different strain, neoCEN-2 and neoCEN-3, which gave less robust CENP-A^Cse4p^ signals, were lost upon sorbose exposure and a new neocentromere, neoCEN-4, appeared at an entirely different locus.

### Neocentromeres Support Stable Chromosome Segregation

Chromosomes carrying a neocentromere exhibited loss rates of ∼10^−4^–10^−5^ per division. This is much more stable than minichromosomes in *S. cerevisiae* (reviewed in [42]) or in *S. pombe* [50] and resembles the high levels of neocentromere stability in humans (reviewed in [6]). Deletion of *CEN5* did not affect cell growth rates unless all Chr5 markers were homozygous, which grew at least as well as *CEN5*-containing strains that were homozygous for all Chr5 markers (Table 1, Figure S1E). Thus, slow growth was due to homozygosis of one or more genes on Chr5 and not to the loss of *CEN5*. *CEN5* deletion occurred with ∼equal frequency on the two Chr5 homologs, suggesting that *C. albicans* requires two different alleles at one or more Chr5 loci to maintain optimal fitness.

The high level of stability in *cen5Δ* strains is consistent with the idea that *C. albicans* has regional centromeres. Yet deletion of *CEN7* was reported previously to result in high levels of Chr7 instability (>50% loss per division) [23]. *CEN7* is the only *C. albicans* centromere lacking any flanking repetitive DNA [22]. The role of repeat DNA in *CEN5* structure, and whether or not it affects centromere function, remains to be determined. However, the presence of repeat DNA at each of the neocentromeres (Figure 8D) suggests that *CEN7* may behave differently from all other *C. albicans* centromeres.

### Neocentromere Formation Is a Highly Efficient Epigenetic Process

A notable property of *C. albicans* neocentromeres is that they form in all *bona fide* transformants. This result is remarkable for two reasons. First, it suggests that when a centromere is perturbed, it induces the formation of neocentromeres. While we cannot rule out that events can occur in the opposite order, our results clearly suggest that loss of a functional centromere stimulates the formation of a new one. Second, it indicates that, when induced to form, neocentromere formation is efficient, since replacement of *CEN5* yields similar numbers of transformants as replacement of a non-essential gene on Chr5. This high level of neocentromere efficiency is unprecedented.

Despite its efficiency, the establishment of neocentromeres was not always rapid. Some transformants appeared early while others continued to appear over time, with high levels of correct transformants appearing as small colonies 7–9 days after transformation. In contrast, when a control gene on Chr5 was disrupted, most colonies appeared within a few days and no new small colonies appeared after several days. Importantly, *C. albicans cen5Δ* strains that initially appeared as small colonies subsequently grew with wild-type growth rate kinetics (Figure 2). This suggests that deletion of a wild-type centromere led to the epigenetic establishment of neocentromeres at new positions and that neocentromere assembly occurred over the first ∼1 week after transformation. Since we isolated similar proportions of proximal and distal neocentromeres from *cen5Δ::URA3* colonies that appeared early or late following transformation, the time at which transformants initially appeared was not a reflection of neocentromere position (e.g., proximal vs distal neoCEN). This slow appearance of transformants is reminiscent of the epigenetic nature of centromere establishment on minichromosomes in *S. pombe*. In this case, cells carrying minichromosomes with functional centromeres were selected because they grew more rapidly than cells carrying minichromosomes that had not yet formed functional centromeres [5].

During the course of this work, Ishii et al. (2008) demonstrated that excision of an *S. pombe* centromere results in several different cell fates. In a large proportion (∼80%) of the cells, the chromosome became unstable, leading to chromosome missegregation and cell death. Cells are rescued at a frequency of <10^−3^ [16] by two types of events: telomere-telomere fusion of the acentric chromosome with one of the other chromosomes or formation of a telocentric neocentromere. Such telomere-telomere fusions were not detected in any of the strains we studied here, although we have documented a telomere-telomere fusion event between Chr5 and an isochromosome (5L) in at least one drug-treated strain [28]. Thus, while telomere-telomere fusions can occur in *C. albicans*, they do not appear to be a major mechanism of chromosome rescue following loss of wild-type centromere DNA.

In *S. pombe*, neocentromere formation was almost completely dependent upon the ability to form heterochromatin. The proportion of rescued cells carrying neocentromeres dropped from ∼75% in wild-type cells to <10% in cells lacking Swi6 (an HP1 homolog), Clr4 (a histone H3 methyl transferase) or Dcr1 (the dicer homolog required for RNAi-dependent heterochromatin formation) [16]. Clearly, *C. albicans CEN5* exhibits epigenetic properties, such as neocentromere formation and movement, in the absence of classic pericentric heterochromatin. Accordingly, we were able to obtain *cen5Δ::URA3* transformants in strains lacking the histone deacetylase ortholog of *S. cerevisiae* Sir2p. The efficiency of transformation and the proportion of Class A to Class B transformants were similar in *sir2Δ/sir2Δ* and isogenic wild-type (*SIR2/SIR2*) *C. albicans* strains (H.W., unpublished data). This result is consistent with the idea that canonical heterochromatin is not necessary for neocentromere formation. We propose that, rather than being required for kinetochore assembly per se, pericentric heterochromatin in larger regional centromeres may be required for the higher order centromere structure necessary to support the organization of multiple microtubule attachment sites per centromere. Because *C. albicans* has only one microtubule attachment per centromere/kinetochore, this type of centromere structure may be dispensable.

### Structural Features of Neocentromeres

The neocentromeres formed in this study had two common features: large intergenic regions and repeated DNA (Figure 8C&D). Similarly, in organisms with larger regional centromeres, centromeres are positioned in ‘gene deserts’. Interestingly, 14 newly evolved centromeres in primate species formed in regions significantly devoid of genes in other primate genomes. [51]. Furthermore, fine structure analysis (at the level of 10–20 kb regions within the ∼2 Mb centromeric domians) of rice centromeres indicates that the very few genes found within a centromeric domain were located within subdomains associated with histone H3 rather than with CENP-A/CEN-H3 histones, suggesting that centromeres evolve from gene-poor regions [51],[52]. Here we found that association with CENP-A^Cse4p^ silences *URA3* in *C. albicans* (Figures 4 & 7). Furthermore, active transcription through centromeric DNA inactivates kinetochore function in *S. cerevisiae* [53] as well as on a human minichromosome [54]. In *S. pombe*, CENP-A^Cnp1^ silences *ura4+* in the centromeric central core in a concentration-dependent manner [55],[56]. We propose that the incompatibility of CENP-A nucleosomes and active transcription is a general feature of centromeres.

The second structural feature of neocentromeres is proximity to repeated DNA sequences. At seven of the eight native centromeres, inverted or direct repeats are found within a few kb of the CENP-A enriched core. Importantly, while a single LTR sequence is sometimes seen within the region, these repeats are different from pericentric repeats flanking larger regional centromeres in other organisms because they are often unique to the individual centromere. For example, the inverted repeat flanking *CEN5* is composed of DNA found only on the two sides of the *CEN5* central core. Similarly, the *GIT1*, *GIT2* and *GIT4* genes are found only in the neoCEN-2 region and orf.6676 and orf19.1124.2 are found only in the neoCEN-3 region (Figure 8D). We propose that a small amount of repeat DNA assists in the assembly of a functional kinetochore in a heterochromatin-independent manner.

### *C. albicans* Neocentromere Locations Can Change

The reversible nature of the change in *URA3* expression status in Class A/proximal neoCEN transformants and the correlated change in CENP-A^Cse4p^ position relative to *URA3* suggests that neocentromeres can move locally, at least in a subset of cells (1/1000) in the population. During selection for chromosome loss on sorbose medium, a poor carbon source, the general telocentric position of the neocentromere in YJB9907 was maintained, although it may have shifted a few kb distal to the telomere. Telocentric chromosomes are common in mice, and *C. albicans CEN6* is telocentric, located ∼50 kb from the Chr6R telomere, indicating that telomeres are one preferred site of centromere assembly, perhaps because they are generally devoid of essential genes and terminate in repetitive DNA.

In strain YJB9929 it appears that the neocentromere(s) were less stable. The presence of two different neocentromere positions YJB9929, which was originally isolated from a single colony, is also consistent with the idea that neocentromere positions are not fixed. Analysis of single colonies suggested that each cell most likely had only one neocentromere on Chr5 (Figure 8B). The smaller size of the neocentromeric DNA region associated with CENP-A^Cse4p^ may result in a smaller number of centromeric nucleosomes than at wild-type centromeres. Human neocentromeres are generally similar in size to normal centromere regions when the size of the constriction is measured by electron microscopy [6]. However, levels of CENP-A at two human neocentromeres, measured with a GFP-CENP-A, are ∼1/3 the level seen at most other centromeres [57], which was proposed to be due to less efficient loading of CENP-A at human neocentromeres [57]. We propose that, in *C. albicans*, the neocentromeres remained functional despite the smaller amount of CENP-A because only one CENP-A nucleosome is required to attach to a single microtubule on each sister chromatid [25].

In contrast to the stable neocentromere in YJB9907s, in two independent YJB9929s strains obtained by sorbose selection for loss of one Chr5 homolog and reduplication of the remaining copy, the neocentromere moved to an entirely new position (neoCEN-4). No CENP-A^Cse4p^ was detected at the neoCEN-4 locus in any of the YJB9907 isolates or in any of the YJB9929 isolates prior to sorbose selection. Thus, upon sorbose selection in YJB9929 a neocentromere formed ∼40 kb from neoCEN-2 on the same chromosome arm or ∼640 kb from neoCEN-3 to the other chromosome arm.

We do not know if neocentromeres are repositioned by sliding along the DNA or by disassembling and reassembling at a new location. We propose that proximal neoCENs reposition by sliding locally (1–2 kb at *URA3*) because they tend to remain close by, resulting in reversible silencing when a neocentromere is within a few kb of *URA3* and not when it is >200 kb away. Furthermore, similar local movements occur at distal neoCENs as evidenced by the slight shift of the neoCEN-1 peak. In contrast, because CENP-A is normally removed from non-centromeric DNA regions [9], it is tempting to speculate that an assembly/disassembly mechanism (Henikoff and Dalal 2005) contributes to distal neoCEN formation as well as to the movement of CENP-A from neoCEN-3 to neoCEN-4 during sorbose selection of strain YJB9929.

### *C. albicans CENs* as a Model for Studying Neocentromere Formation and Movement

*C. albicans* centromeres are unique in that neocentromere formation at multiple positions is efficiently induced by deletion of a centromere. As in humans and Drosophila, chromosome breakage and healing (mediated by homologous recombination between *URA3* and the *CEN5* flanking DNA) accompanies neocentromere formation events in *C. albicans cen5Δ* transformants. This suggests that, while similar mechanisms operate in organisms that have pericentric heterochromatin, the process is simpler and much more efficient in *C. albicans* because the complexity of heterochromatin does not come into play.

Neocentromeres can form close to the site of the original centromere (as in Drosophila [15]), at distal loci with no sequence conservation (as in humans (reviewed in [6]) or near a telomere (as in *S. pombe* [16])). In humans, there are a small number of cases in which neocentromeres appear at ectopic positions and are not associated with any obvious chromosome rearrangement (reviewed in [6],[58]). While the formation of neocentromeres in *C. albicans* followed deletion of the wild-type centromere, we did not detect new aneuploidies or translocations in any of these strains. This is especially interesting in light of the high levels of chromosome rearrangements that can occur in *C. albicans* strains [59]. Another unique aspect of *C. albicans* centromeres is that the movement of proximal neoCENs can be monitored by selection and counter-selection for *URA3*. This provides an extraordinarily powerful tool for direct studies of the mechanisms of local neocentromere movement and demonstrates that, while rare, short range neocentromere movement (several kb) is more frequent than movement over much longer distances.

There is much that we do not understand about regional centromeres. In part, this is because *S. cerevisiae* point centromeres cannot be used to study epigenetic phenomena, such as neocentromere formation and movement, that occur only at regional centromeres. The small size and simplicity of *C. albicans* regional centromeres greatly facilitates studies of the core functions of regional centromeres without the complications associated with long, complex repeat tracts and pericentric heterochromatin. In this initial study, we found that *C. albicans* centromeres form neocentromeres, despite having no pericentric heterochromatin. Furthermore, neocentromeres can form at multiple chromosomal loci including loci close to the deleted centromere, telomeric loci and other distal loci on both chromosome arms. Finally, neocentromeres can move over short or long distances to new loci and can silence a gene associated with it. Additional work will be needed to identify the environmental and genetic conditions that influence the rate of formation as well as the structural features that modulate the location and movement of neocentromeres.

## Materials and Methods

### Strain Construction and Analysis of Transformants

Strains are listed in Table S1 and were constructed using PCR-mediated gene deletion with 70 nucleotides of homology to genomic DNA with gene disruption primers listed in Table S2 and a lithium acetate protocol [60]. *CEN5* was deleted by amplification of *URA3* from pGEM-*URA3* [60], which was inserted at *CEN5* in both orientations. As an alternate approach, *CEN5* was replaced with *NAT1* from plasmid pMG2120 using primers 3161 and 3162. Strain YJB9779 (5L-*URA3*-Tel) was constructed by transforming strain YJB7617 with *URA3-TEL* amplified from pMG2192 using primers 2249 and 1489 to insert it 360 bp to the right of the IR (37 bp centromere proximal to the orf19.4219 coding sequence). pMG2192 was constructed by digesting pMM100 [61] with *Not*1*/Xho*1 and 1000 bp CA7 telomere repeat was ligated to *Xho*1/*Not*1 cut pYPB1-*ADH1* [62]. Strain YJB9858 (TEL-*NAT1*-5R) was constructed by transforming YJB7617 with TEL-*NAT1* amplified from pMG2194 using a forward primer (2313) that produces a fragment that can insert 1 kb to the left of the IR (660 bp centromere proximal to the orf19.3161 coding sequence) and a reverse primer (1489) with homology to telomere sequence in the plamid. pMG2194 was constructed by digesting pMM100 [61] with *Apa*I and the 900 bp fragment containing the telomeric repeat was cloned into pMG2171 [29]. *C. albicans* transformants were isolated after incubation on SDC-uri or YPAD+Nat at 30°C.

Transformation frequencies and the proportion of *bona fide* transformants was similar for *cen5Δ* and control strains. The frequency of insertion into the long and short *CEN5* homologs was also similar when either earlier or later appearing transformants were analyzed. For example, for a *cen5Δ::URA3* transformation, of the 61 transformants, 8 were correct insertions and 4 deleted *CEN5* long and 4 deleted *CEN5* short. Transformants were screened for those with *bona fide* insertions by PCR using primers (Table S2) flanking the insertion site together with primers within the inserted DNA as illustrated in Figure S1A. The *CEN5* homolog that was disrupted with *URA3* was identified by Southern analysis of conventional and CHEF karyotype gels using methods previously described [28]. Probes were amplified with primers listed in Table S2.

### CHEF Gel Analysis and Comparative Genome Hybridization Arrays

*C. albicans* karyotypes were analyzed by CHEF gel electrophoresis as described previously [33] using conditions that optimize visualization of chromosomes 2–7. Comparative genome hybridization (CGH) was performed as described [33] and was plotted to the *C. albicans* genetic map using an updated version of the Chromosome_Map [33], based on Assembly 21 coordinates [63],[64].

### Southern Hybridization

DNA was transferred from agarose gels to Magnacharge nylon membranes (GE Osmonics, Minnetonka, MN) [65]. Membranes were probed overnight at 42°C and detected with anti-Digoxigenin-Alkaline Phosphatase and CDPstar essentially as described [66]. Probes were prepared by PCR amplification using DIG-labelled nucleotides according to manufacturer's instructions (Roche, Indianapolis, IN).

### Sorbose-Derived cen5Δ::URA3/cen5Δ::URA3 Strains

Strains carrying *cen5Δ::URA3* were grown on sorbose medium without added uridine for 7–9 days. Colonies that appeared were streaked to YPAD media and large colonies that appeared early were then streaked to SDC-uri medium to ensure that they had retained the *URA3* marker. PCR analysis was performed to identify derivative strains in which the *cen5Δ::URA3* fragment was retained, the wild-type *CEN5* was no longer detectable, and that only one of the two *MTL* loci on Chr5 was retained, using primers listed in Table S2.

### Growth Rate Analysis

Growth rates were determined using overnight stationary phase cultures diluted 1∶10^5^ in YPAD media covered with mineral oil in a 96-well plate (Corning, NY). Cultures were incubated in a microplate spectrophotometer (Sunrise, Tecan, San José, CA) at 30°C with constant linear shaking. OD readings were taken every 15 min. Doubling time was calculated based on the exponential constant of the fitted exponential curve of each well using software kindly provided by Sven Bergmann and Gil Hornung.

### Mitotic Stability and Fluctuation Assays of Chromosome Loss Rates

Mitotic stability was determined by comparing the proportion of cells containing the relevant marker (*URA3* or *NAT1*) relative to the total number of cells in the population following growth under conditions selective for the marker [67]. Fluctuation analysis of loss rates was performed as described [39] using the method of the median [40]. Briefly, strains were streaked for single colonies and grown on SDC-uri for 2 days at 30°. Twenty independent colonies per strain were inoculated into 5 ml liquid non-selective medium (YPAD) and grown overnight at 30°C with shaking. Cultures were harvested by centrifugation and washed twice in 1 ml of sterile water. Appropriate dilutions were plated onto nonselective YPAD for total cell counts and selective media (SD+FOA) for selection of Ura- colonies. Plates were incubated at 30°C for 2–3 days, and colony counts were used to calculate the rate of FOA^R^/cell division [39]. To distinguish loss of the *URA3* marker from silencing, we analyzed FOA resistant colonies identified in loss rate fluctuation analyses done with strain YJB10064 (*his1::NAT1/HIS1* ∼38 kb from the Chr5R telomere) in which *either CEN5 or MYO1* had been replaced *with URA3*. The resulting strains [YJB10169 (*myo1*Δ*::URA3*), YJB10233 (*cen5*Δ*::URA3* Class A), and YJB10234(*cen5*Δ*::URA3* Class B)] were replica-plated to SDC-his, SDC-uri, YPAD+Nat, and YPAD. FOA^R^ colonies that re-grew when replica plated to SDC-uri were candidates for *URA3* silencing. Images of the SDC-his, YPAD+Nat, and YPAD replica plates were overlaid in order to classify colonies as Nat^R^His^+^, Nat^S^His^+^, or Nat^R^His^−^, using the YPAD replica plate as a control for colony transfer. From each colony class obtained per strain, five colonies were analyzed by PCR amplification of the *MTL* loci using primers 1193, 1194, 1195 and 1197 (Table S2).

### CENP-A^Cse4p^ Antibody Production

Rabbit polyclonal anti-CENP-A^Cse4p^ antibodies were raised against an N-terminal peptide (amino acids 1–18 (Ac-MARLSGQSSGRQTGQGTSC-amide)) of Ca CENP-A^Cse4p^ and affinity purified (Quality Controlled Biochemicals; Hopkinton, MA) following the protocol of Sanyal and co-workers [68].

### Chromatin Immunoprecipitation

ChIP was performed using extraction protocols modified from Weber et al. [69] and Carbon and Co-workers [23]. Overnight cultures were diluted to an OD600 of ∼0.3 and harvested at an OD600 of ∼1.0. Cells were fixed in 1% formaldehyde for 15 min at 30°C and then treated with zymolyase (0.2 mg/ml+0.5 mM AEBSF) until 80–90% of cells were spheroplasts. Reactions were stopped with 1.2 M Sorbitol, 1 mM MgCl_2_, 20 mM PIPES pH 6.8, and sonicated to yield DNA fragments of 500–600 bp. Input lysate was collected after insoluble matter was precipitated twice by centrifugation. The lysate was incubated at 4°C for ∼16 h with or without 4 µg/ml anti-CENP-A^Cse4p^ antibody. Antibody and associated DNA was incubated with Protein A-agarose beads at 4°C for ≥3 hrs, washed several times and then eluted twice at 65°C. Crosslinks were reversed overnight at 65°C followed by RNase A and proteinase K treatments. Proteins were removed by phenol-chloroform/isoamyl alcohol/chloroform extraction. Samples were ethanol precipitated, washed in 70% ethanol and resuspended in 80 ml TE.

Each Class A ChIP experiment was performed with the same isolate grown in SDC-uri and in SD+FOA. ChIP experiments were repeated with 4 different Class A strains (YJB9909, YJB9915, YJB9916 and YJB9926) analyzing 3 independent colonies from YJB9909 and YJB9915 and one colony each from strains YJB9916 and YJB9926. Two independent colonies from each of two different Class B strains (YJB9907 and YJB9861) also were analyzed. Contiguous fragments spanning ∼15 kb across *CEN5* were amplified using primer pairs listed in Table S2. Dilution PCRs were used to ensure that the amplification of each PCR product was in the linear range. PCR products were run on an 1.4% agarose gel and band intensities were quantified using ImageJ software (NIH) with a background correction macro. Intensities were calculated (+Ab/lysate) and normalized to the mean intensity of *CEN5* central core PCR products in all strains except *cen5Δ/cen5Δ* strains, in which case it was normalized to the mean intensity of *CEN4* central core products. “No-antibody” samples were run in parallel to ensure quality of the samples and reactions (e.g., Figure 6). Correlation coefficients for groups of samples were performed using the Excell ‘Correlation’ function [70].

### ChIP-SEQ by Massively Parallel High-Throughput Sequence Analysis

Chromatin lysates and samples immunoprecipiated with CENP-A^Cse4p^ antibody (as described above) were processed for massively parallel high throughput sequencing using the Illumina Genome Analyzer Classic following manufacturer's instructions. Samples were nebulized to an average ∼200 bp fragment size. The ends were repaired and an “A” base was added to the 3′ ends. Illumina adapter oligos were ligated to the fragments and these were purified on a 2.0% agarose gel by excising a region of the gel corresponding to 200 base pairs. After purification using a Qiagen Gel Extraction kit,(Qiagen, Valencia, CA), the adapter modified DNA fragments were enriched by PCR. The enriched fragments were purified using a Minelute PCR purification kit from Qiagen and quantified using the Quant-iT dsDNA HS Assay Kit from Invitrogen (Carlsbad, CA). The samples were then diluted to give a final concentration of 2 pM and cluster generation was performed on the Illumina Cluster Station (Illumina, San Diego, CA) following manufacturer's instructions. The resulting flow cell was sequenced on the Illumina GA Classic for 36 cycles.

The sequences were extracted from .*tiff* image files using *Firecrest* and *Bustard* tools of the Illumina Genome Analyzer pipeline. These sequence reads were aligned to the human genome as reference sequence (NCBI v36.49 from ensembl) using Illumina's *ELAND* tool. Both 36 bp and 25 bp reads were used, along with unmasked and repeat-masked [71] human genome. Peaks in the read coverage from uniquely aligned reads were identified using the *FindPeaks* tool [72]. Release version 3.1 of the tool was used with default parameters for binding site search, except the values for *subpeaks* as 0.2 and *trim* as 0.2. For non-overlapping sliding window bins of chromosome 5 data, we calculated the ratio of reads mapped (aligned) in the bin, to the total number of reads mapped on the chromosome. The difference of this ratio in the sample compared to control was calculated to identify enriched bins along the chromosome for that sample.

### Quantitative Real-Time PCR (qRT-PCR)

RNA was extracted from cells grown to log phase in SDC-uri or SD+FOA using the Epicentre MasterPure yeast RNA purification kit (Epicentre Biotechnologies, Madison, WI) according to manufacturer's instructions. cDNA was prepared with the BioRad iScript cDNA Synthesis Kit (Bio-Rad Laboratories, Hercules, CA) with a combination of random hexamers and oligo (dT) primers. Real-time PCR was performed using the Roche LightCycler FastStart DNA Master^PLUS^ SYBR Green I kit (Roche Applied Science, Indianapolis, IN). Reactions were run on the Eppendorf Mastercycler ep realplex machine (Eppendorf, Westbury, NY). Primers used are listed in Table S2. A no RT negative control and melt curve analysis were performed on each run to ensure that no contaminating DNA or second products were amplified. Duplicate wells and 3 technical repeats were performed on cDNA from *cen5Δ::URA*3 Class A strains YJB9909 and YJB9916. ΔC_t_ values were determined from the mean of the results of all technical and biological replicates (Figure 4C). Error bars show standard error of the mean (SEM).

## Supporting Information

Figure S1 Analysis of *cen5Δ::URA3* transformants. A. PCR analysis of *cen5Δ::URA3* transformants. Location of primer sets that amplify left (L) and right (R) borders of wild-type *CEN5* and *cen5Δ::URA3* alleles. Left and Right reactions each contained 3 primers (A (2002), B (1961), C (945) or X (1915),Y (1901), Z(944)). Primer sequences are listed in Table S2. Expected amplified fragment sizes from wild-type (L2, R2) and from the *cen5Δ::URA3* allele (L1, R1) are indicated. Correct *cen5Δ::URA3* transformants contain all four PCR products. B. PCR analysis of *cen5Δ::NAT1* transformants. Location of primer sets that amplify left (L) and right (R) borders of wild-type *CEN5* and *cen5Δ::NAT1* alleles. Lanes 1, primers A+B; lanes 2, primers A+W; lanes 3, primers X+Y; lanes 4, primers V+X; lanes 5, primers A+B+W; and lanes 6, primers V+X+Y. Wild-type strain BWP17 was used as a negative control. C. Southern analysis of *cen5Δ::NAT1* transformants. As in Figure 1C, DNA from *cen5Δ::NAT1* (lane 1, YJB10805; lane 2, YJB10828) was digested with *EcoRV* and *Sac*II, separated by agarose gel electrophoresis and probed for *NAT1*. The diagram indicates the position of restriction sites, expected sizes of restriction fragments, and probe locations. *NAT1* was inserted on the short homolog in one strain (YJB10828), in the long homolog in the other (YJB10805). D. CHEF gel analysis of *cen5Δ::NAT1* strains reveals no major karyotype alterations. Lane 1, *cen5Δ::NAT1* (YJB10805); lane 2, *cen5Δ::NAT1* (YJB10828). E. Growth curve analysis of strains homozygous for chromosome 5. Wild-type (RM10, black line) and sorbose-derived strains lacking *CEN5* (YJB9907-6s, green; and YJB9907-3s, pink) or with intact *CEN5* (YJB9726, cyan) were grown at 30 C. Note that number of cells in the initial culture influences the time when logarithmic division begins and differs between experiments; slope of the logarithmic phase of all curves was not significantly different between experiments (see Table 1).
(0.8 MB PDF)

Figure S2 Chromatin immunoprecipitation of *CEN5* and *cen5Δ::URA3* DNA. A. Chromatin immunoprecipitation of the wild type homolog of Class A long *cen5*Δ strains. Contiguous ChIP PCR of *CC5* and the flanking IR displayed as in Figure 7A for *cen5Δ::URA3* strains that disrupted the long allele of *CEN5*. Class A strains were grown in SDC-uri (white) and then selected on SD+FOA (Gray). Class B strains were grown only on SDC-uri. Class A long allele strains represent the average of 4 extracts (two from YJB9916 and two from YJB9926). Class B long allele extracts were made from YJB9907 (two extracts) and YJB9929 (one extract). B. Chromatin immunoprecipitation of the wild type homolog of Class A short *cen5Δ* strains. Contiguous ChIP PCR of *CC5* and the flanking IR displayed as in Figure 7A for *cen5Δ::URA3* strains that disrupted the short (B) allele of *CEN5*. Class A strains were grown in SDC-uri (white) and then selected on SD+FOA (Gray). Class B strains were grown only on SDC-uri. Class A short allele strains represent the average of 4 extracts (two from YJB9909 and two from YJB9915). Class B short allele extracts were made from YJB9861 (two extracts). As in wild-type cells, CENP-A^Cse4p^ specifically associates with *CC5*. The LTR within *CC5* (gray triangle) is detected only in extracts from strains in which the short homolog was deleted and the long (LTR-containing) homolog is retained. C. Chromatin immunoprecipitation of Class A long allele under selection and counterselection for *URA3* expression. CENP-A^Cse4p^ associates with the LTR to the left of *CEN5* when *cen5Δ::URA3* has replaced *CEN5* in the long allele (YJB9916). A representative contiguous ChIP PCR of *cen5Δ::URA3* displayed as in Figure 7B but including the *episemon* LTR present on the long allele of *CEN5* which was replaced with *URA3* in strain YJB9916. ChIP was performed with cells grown in SDC-uri (white) or selected on 5-FOA (black). Similar results were obtained with YJB9926. D. Chromatin immunoprecipitation of Class B *cen5Δ* strains. CENP-A^Cse4p^ does not associate with *URA3* or flanking DNA when Class B transformants are grown in SDC-uri. Contiguous ChIP PCR of Class B *cen5D::URA3* strains (YJB9861 (white) and YJB9907 (black)) grown in SDC-uri, normalized and displayed as in Figure 7A.
(0.8 MB PDF)

Figure S3 Centromeric DNA regions associated with CENP-A^Cse4p^. Chromosome regions detected by ChIP-SEQ analysis of wild-type strain RM10 (red line) are compared, for each chromosome (listed at left) to regions previously reported as *C. albicans* centromere DNA, based on ChIP with primers spanning ∼1 kb regions of the genome [22] (blue line). Lines are drawn to scale, chromosome coordinates for the regions are indicated at the ends of the lines and the length of the DNA associated with CENP-A^Cse4p^ at each centromere (in bp) is indicated to the right. Chr5 coordinate numbers also correspond to the position of the ChIP-SEQ peak in Figure 8B.
(0.05 MB PDF)

Figure S4 PCR analysis of neoCEN regions in Class B strains. Regions analyzed for strains indicated on left are within the peaks identified in Figure 8B. Chromosomal coordinates and positions of the PCR products relative to ORFs (black) arrows are diagramed and primer pairs used are listed in Table S1. A. Analysis of YJB9907 and its derivatives. Top, YJB9907; bottom, *cen5Δ::URA3* homozygous strains YJB9907-3s (grey bars) and YJB9907-6s (black bars). No *CEN5* DNA is present in these strains. B. Analysis of YJB9929 and its derivatives. Top panel, YJB9907; middle panel, single colony #4 from YJB9929 grown in rich medium; Bottom panel, *cen5Δ::URA3* homozygous strains YJB9929-1s (grey bars) and YJB9929-2s (black bars). No *CEN5* DNA is present in these strains.
(0.1 MB TIF)

Table S1 Strains used in this study.
(0.1 MB DOC)

Table S2 Primers used in this study.
(0.2 MB DOC)
