# Supplementary figures and images for: Neocentromeres Form Efficiently at Multiple Possible Loci in Candida albicans
Source: PLoS Genet. 2009 Mar 6;5(3):e1000400. doi: 10.1371/journal.pgen.1000400 (PMC2642679; doi:10.1371/journal.pgen.1000400)

A

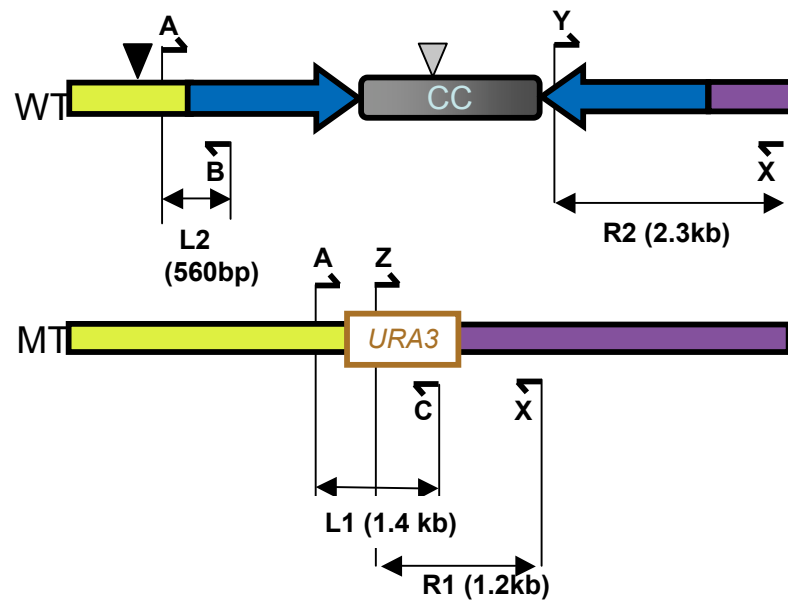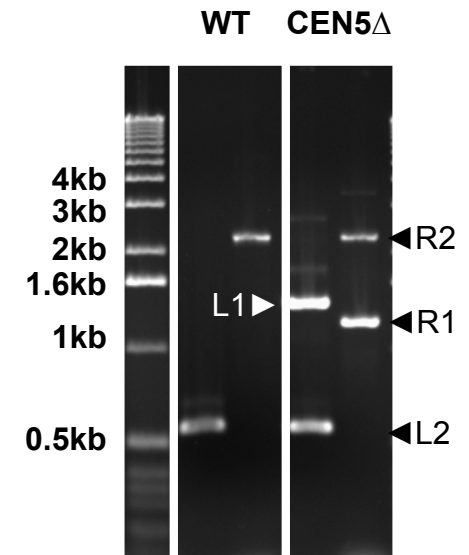

B

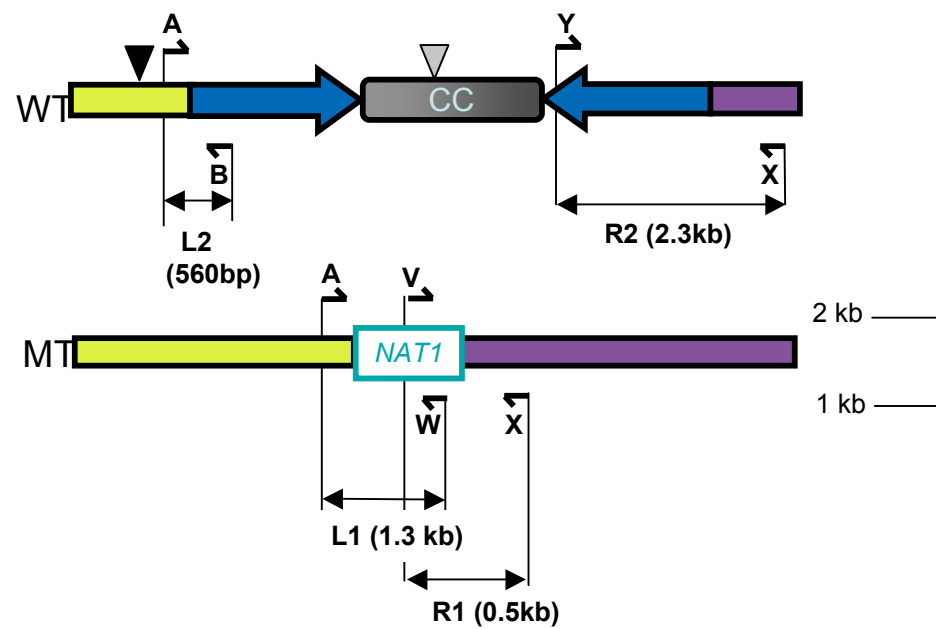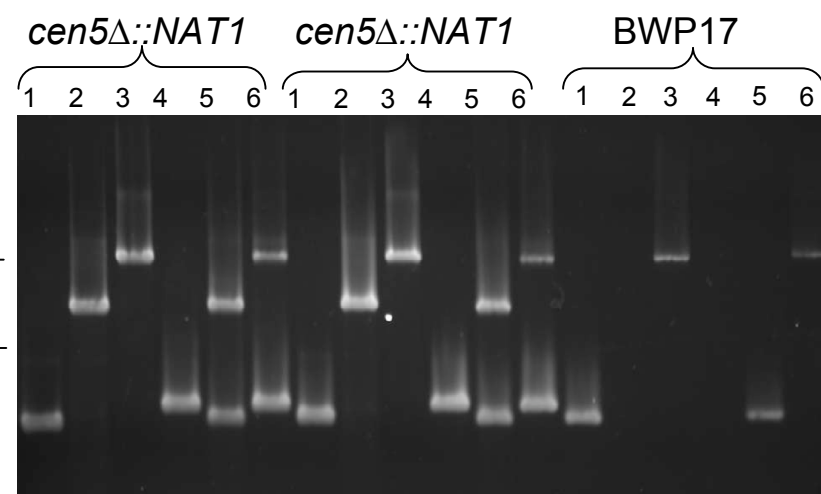

C

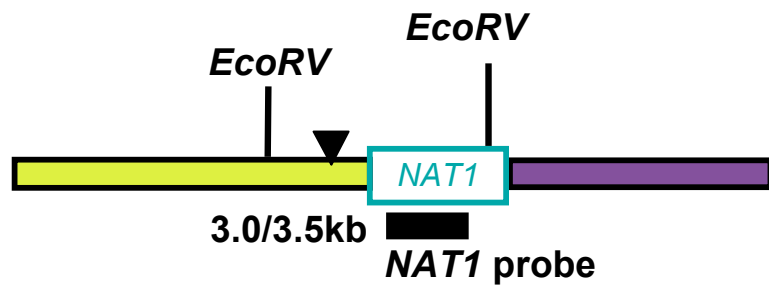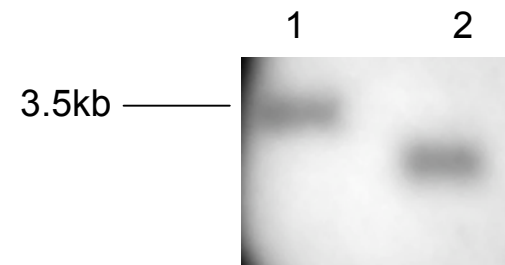

D

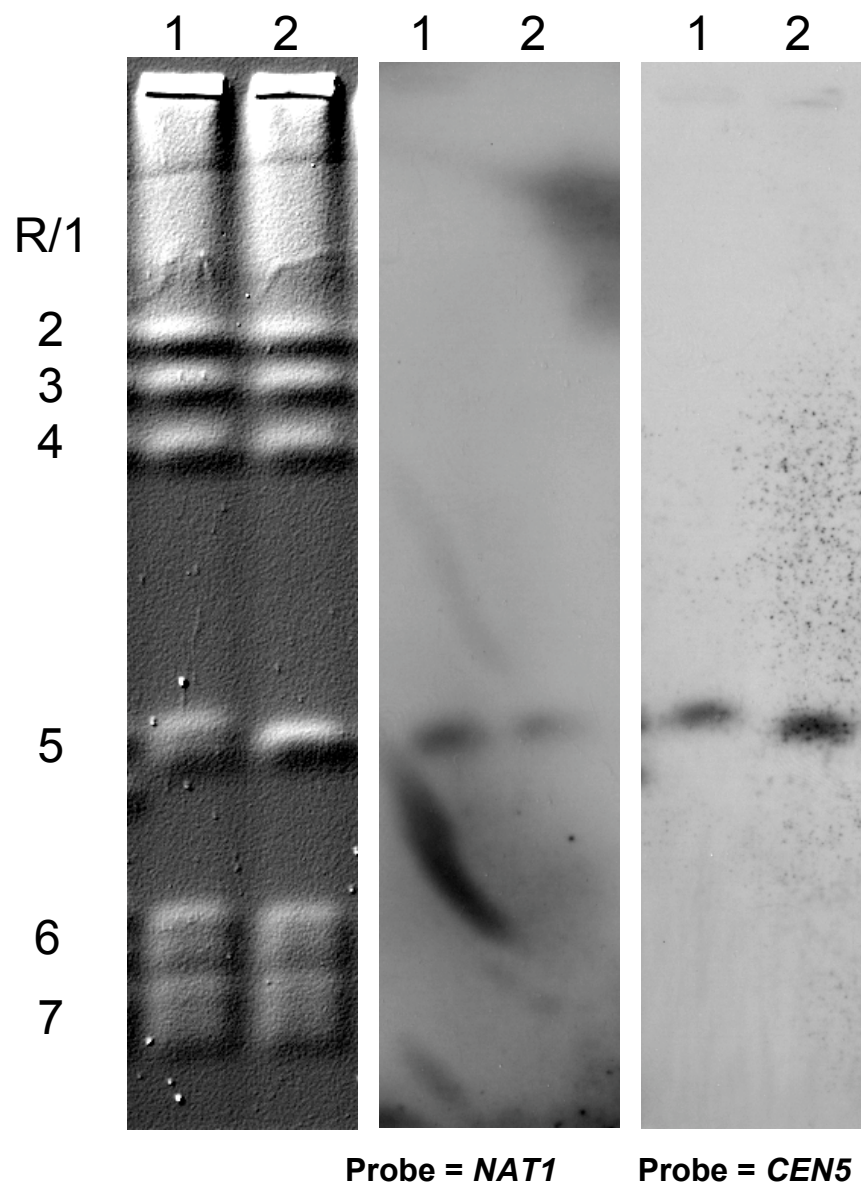

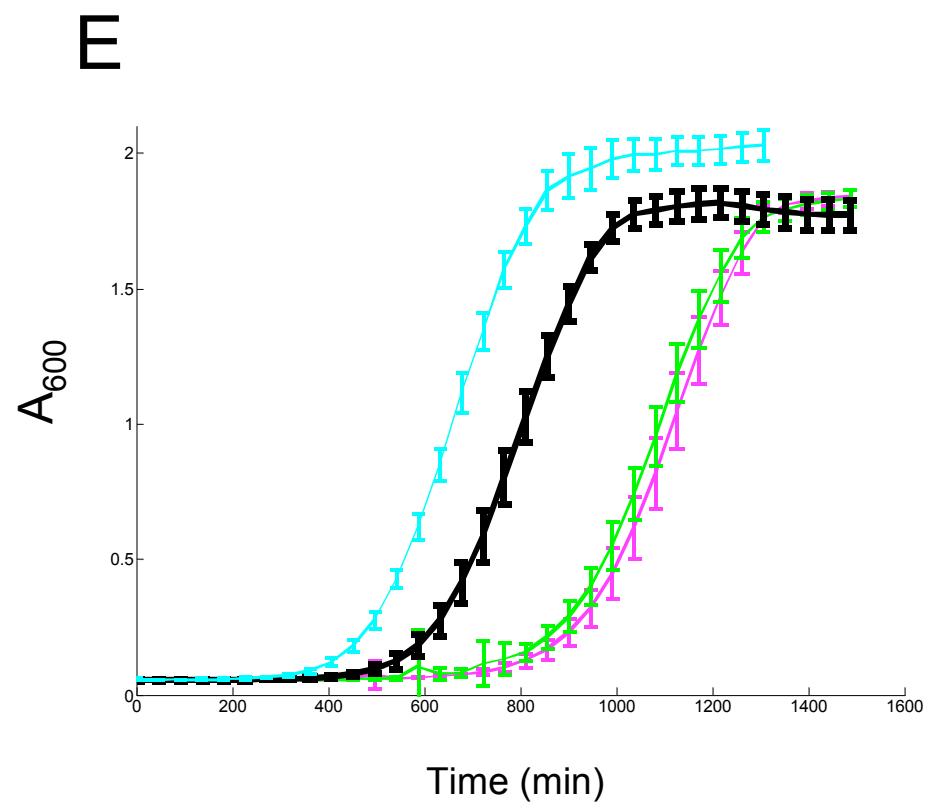

Supplement: Figure S1 — Analysis of cen5Δ::URA3 transformants. A. PCR analysis of cen5Δ::URA3 transformants. Location of primer sets that amplify left (L) and right (R) borders of wild-type CEN5 and cen5Δ::URA3 alleles. Left and Right reactions each contained 3 primers (A (2002), B (1961), C (945) or X (1915),Y (1901), Z(944)). Primer sequences are listed in Table S2. Expected amplified fragment sizes from wild-type (L2, R2) and from the cen5Δ::URA3 allele (L1, R1) are indicated. Correct cen5Δ::URA3 transformants contain all four PCR products. B. PCR analysis of cen5Δ::NAT1 transformants. Location of primer sets that amplify left (L) and right (R) borders of wild-type CEN5 and cen5Δ::NAT1 alleles. Lanes 1, primers A+B; lanes 2, primers A+W; lanes 3, primers X+Y; lanes 4, primers V+X; lanes 5, primers A+B+W; and lanes 6, primers V+X+Y. Wild-type strain BWP17 was used as a negative control. C. Southern analysis of cen5Δ::NAT1 transformants. As in Figure 1C, DNA from cen5Δ::NAT1 (lane 1, YJB10805; lane 2, YJB10828) was digested with EcoRV and SacII, separated by agarose gel electrophoresis and probed for NAT1. The diagram indicates the position of restriction sites, expected sizes of restriction fragments, and probe locations. NAT1 was inserted on the short homolog in one strain (YJB10828), in the long homolog in the other (YJB10805). D. CHEF gel analysis of cen5Δ::NAT1 strains reveals no major karyotype alterations. Lane 1, cen5Δ::NAT1 (YJB10805); lane 2, cen5Δ::NAT1 (YJB10828). E. Growth curve analysis of strains homozygous for chromosome 5. Wild-type (RM10, black line) and sorbose-derived strains lacking CEN5 (YJB9907-6s, green; and YJB9907-3s, pink) or with intact CEN5 (YJB9726, cyan) were grown at 30 C. Note that number of cells in the initial culture influences the time when logarithmic division begins and differs between experiments; slope of the logarithmic phase of all curves was not significantly different between experiments (see Table 1). (0.8 MB PDF) [file pgen.1000400.s001.pdf]

A

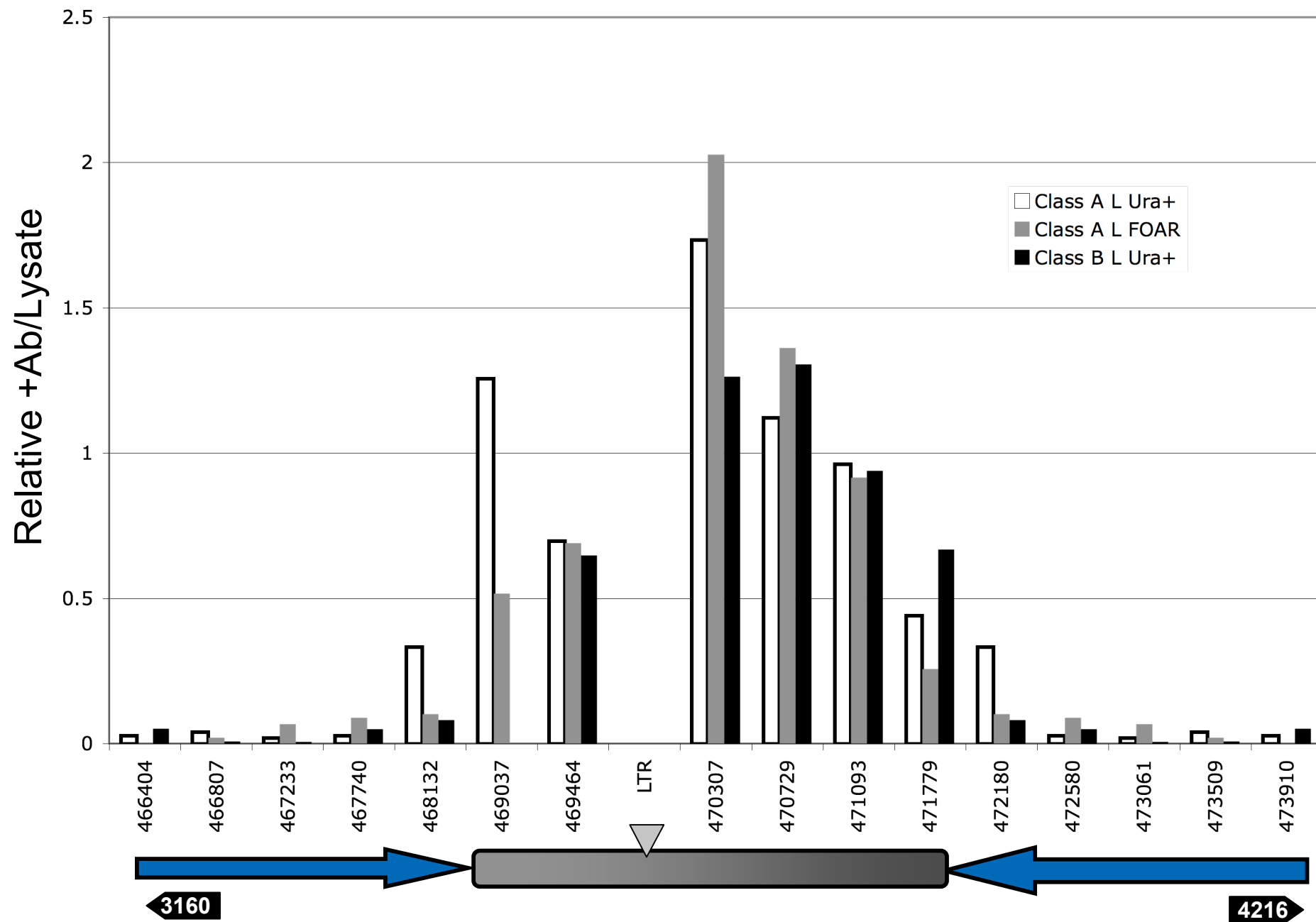

**B**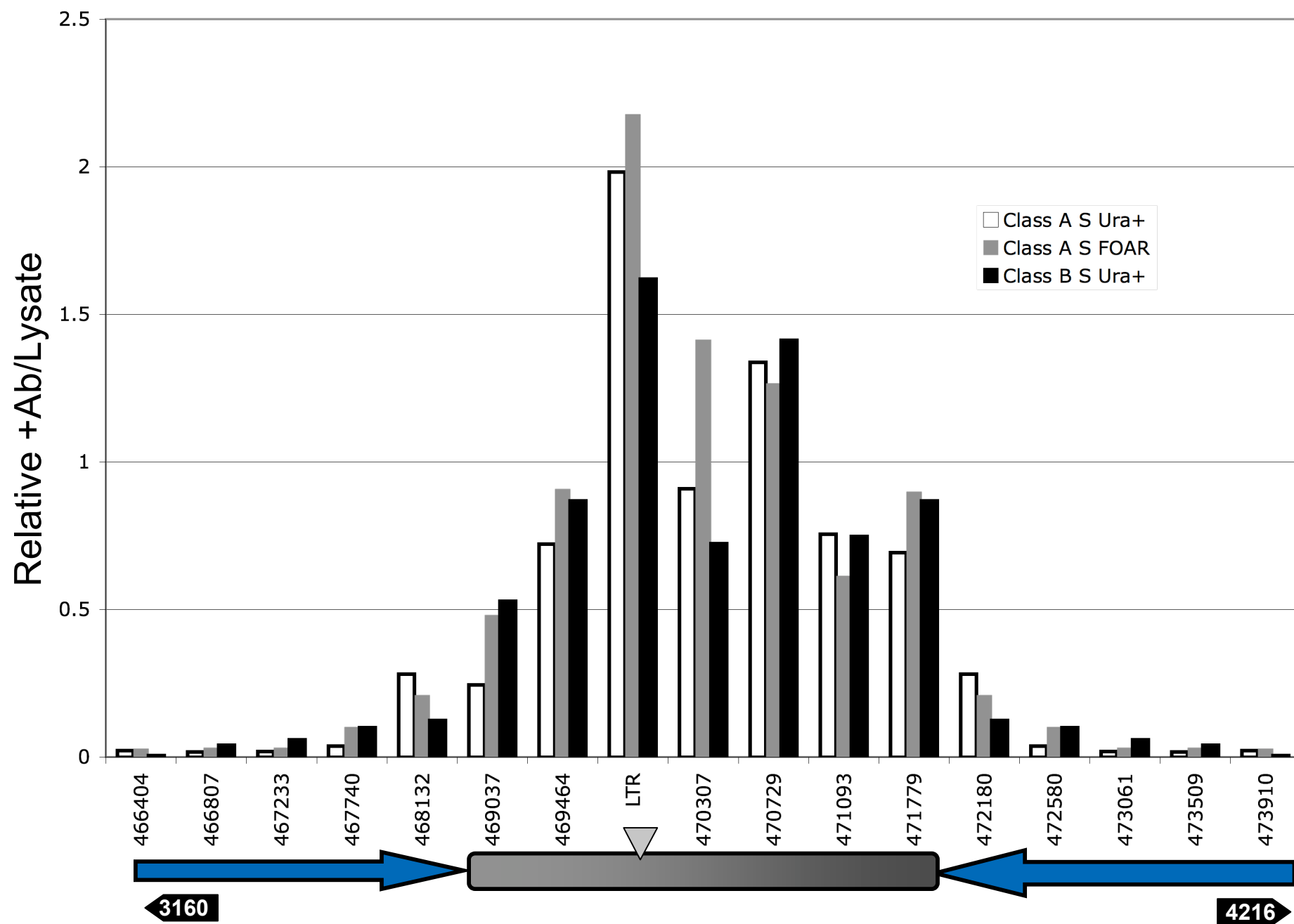

C

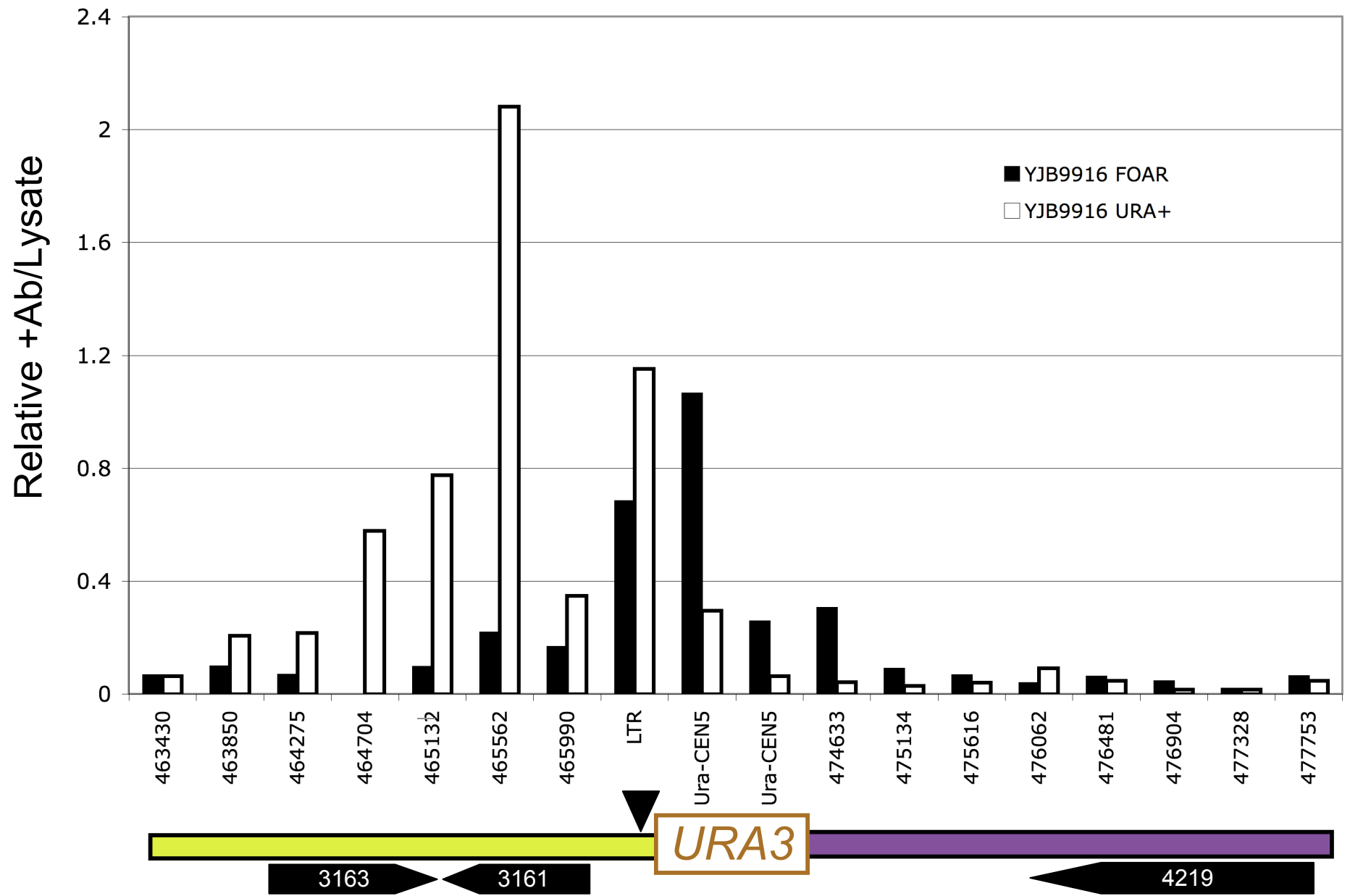

D

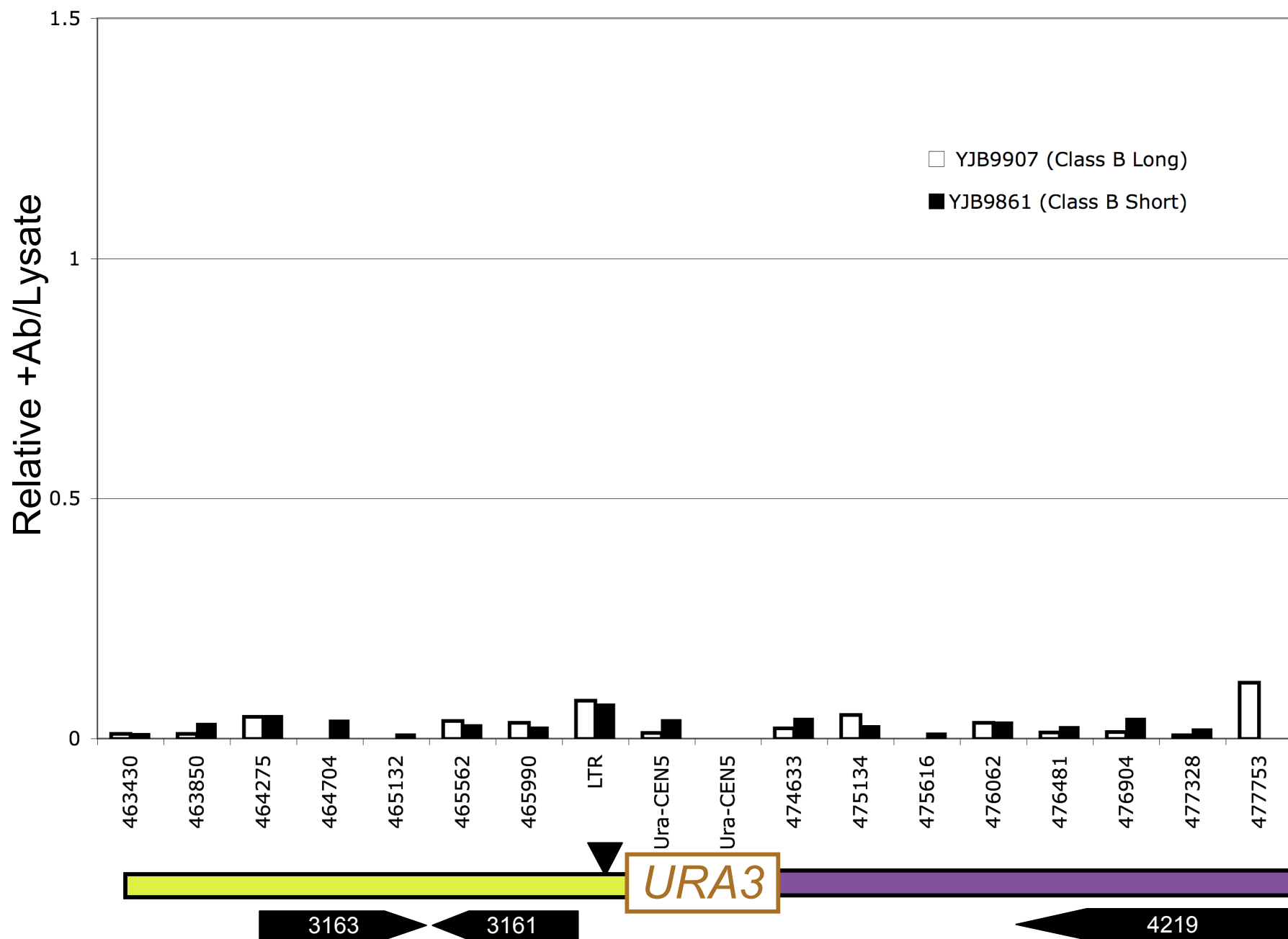

Supplement: Figure S2 — Chromatin immunoprecipitation of CEN5 and cen5Δ::URA3 DNA. A. Chromatin immunoprecipitation of the wild type homolog of Class A long cen5Δ strains. Contiguous ChIP PCR of CC5 and the flanking IR displayed as in Figure 7A for cen5Δ::URA3 strains that disrupted the long allele of CEN5. Class A strains were grown in SDC-uri (white) and then selected on SD+FOA (Gray). Class B strains were grown only on SDC-uri. Class A long allele strains represent the average of 4 extracts (two from YJB9916 and two from YJB9926). Class B long allele extracts were made from YJB9907 (two extracts) and YJB9929 (one extract). B. Chromatin immunoprecipitation of the wild type homolog of Class A short cen5Δ strains. Contiguous ChIP PCR of CC5 and the flanking IR displayed as in Figure 7A for cen5Δ::URA3 strains that disrupted the short (B) allele of CEN5. Class A strains were grown in SDC-uri (white) and then selected on SD+FOA (Gray). Class B strains were grown only on SDC-uri. Class A short allele strains represent the average of 4 extracts (two from YJB9909 and two from YJB9915). Class B short allele extracts were made from YJB9861 (two extracts). As in wild-type cells, CENP-ACse4p specifically associates with CC5. The LTR within CC5 (gray triangle) is detected only in extracts from strains in which the short homolog was deleted and the long (LTR-containing) homolog is retained. C. Chromatin immunoprecipitation of Class A long allele under selection and counterselection for URA3 expression. CENP-ACse4p associates with the LTR to the left of CEN5 when cen5Δ::URA3 has replaced CEN5 in the long allele (YJB9916). A representative contiguous ChIP PCR of cen5Δ::URA3 displayed as in Figure 7B but including the episemon LTR present on the long allele of CEN5 which was replaced with URA3 in strain YJB9916. ChIP was performed with cells grown in SDC-uri (white) or selected on 5-FOA (black). Similar results were obtained with YJB9926. D. Chromatin immunoprecipitation of Class B cen5Δ strains. CENP-A [file pgen.1000400.s002.pdf]

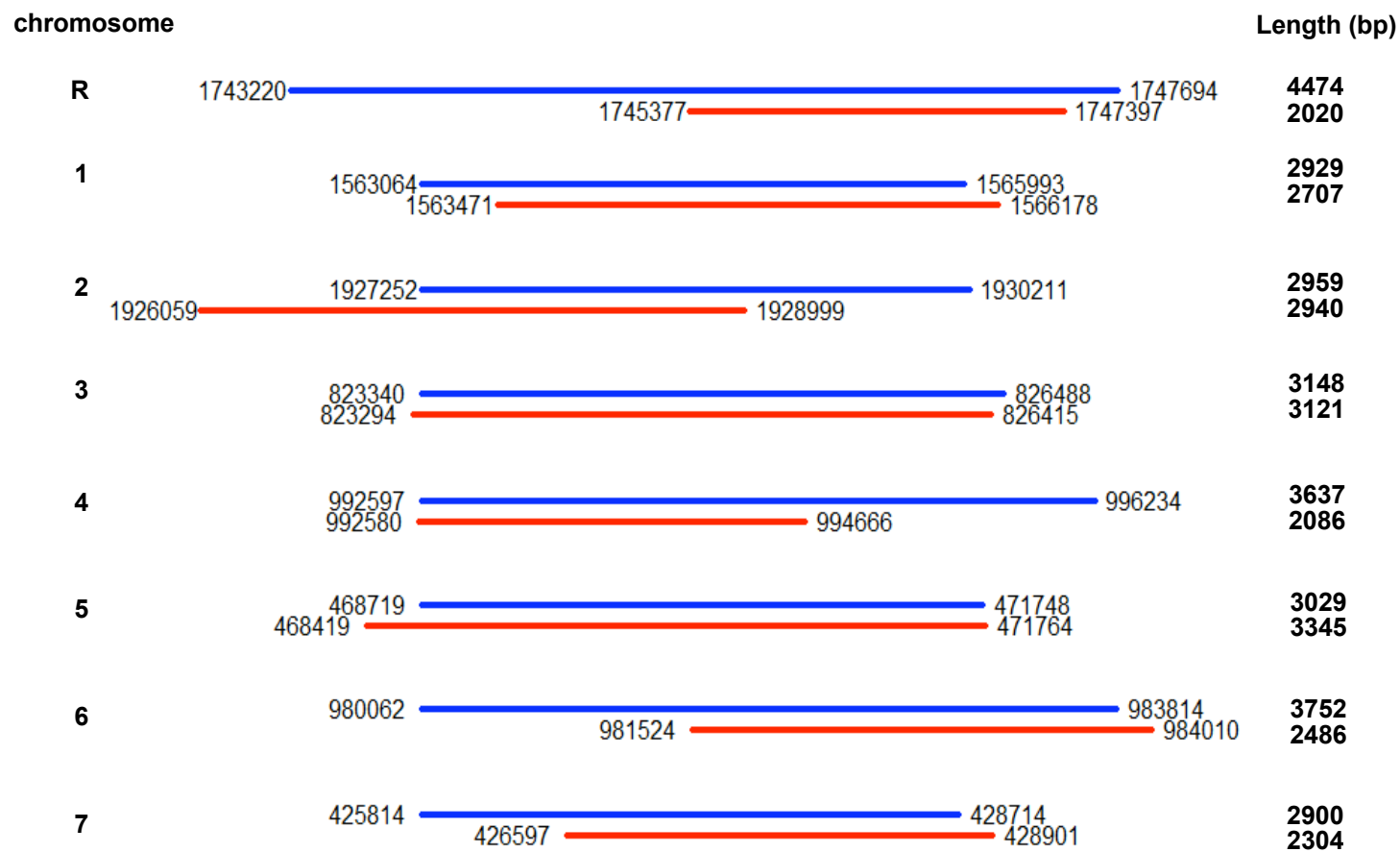

Supplement: Figure S3 — Centromeric DNA regions associated with CENP-ACse4p. Chromosome regions detected by ChIP-SEQ analysis of wild-type strain RM10 (red line) are compared, for each chromosome (listed at left) to regions previously reported as C. albicans centromere DNA, based on ChIP with primers spanning ∼1 kb regions of the genome [22] (blue line). Lines are drawn to scale, chromosome coordinates for the regions are indicated at the ends of the lines and the length of the DNA associated with CENP-ACse4p at each centromere (in bp) is indicated to the right. Chr5 coordinate numbers also correspond to the position of the ChIP-SEQ peak in Figure 8B. (0.05 MB PDF) [file pgen.1000400.s003.pdf]

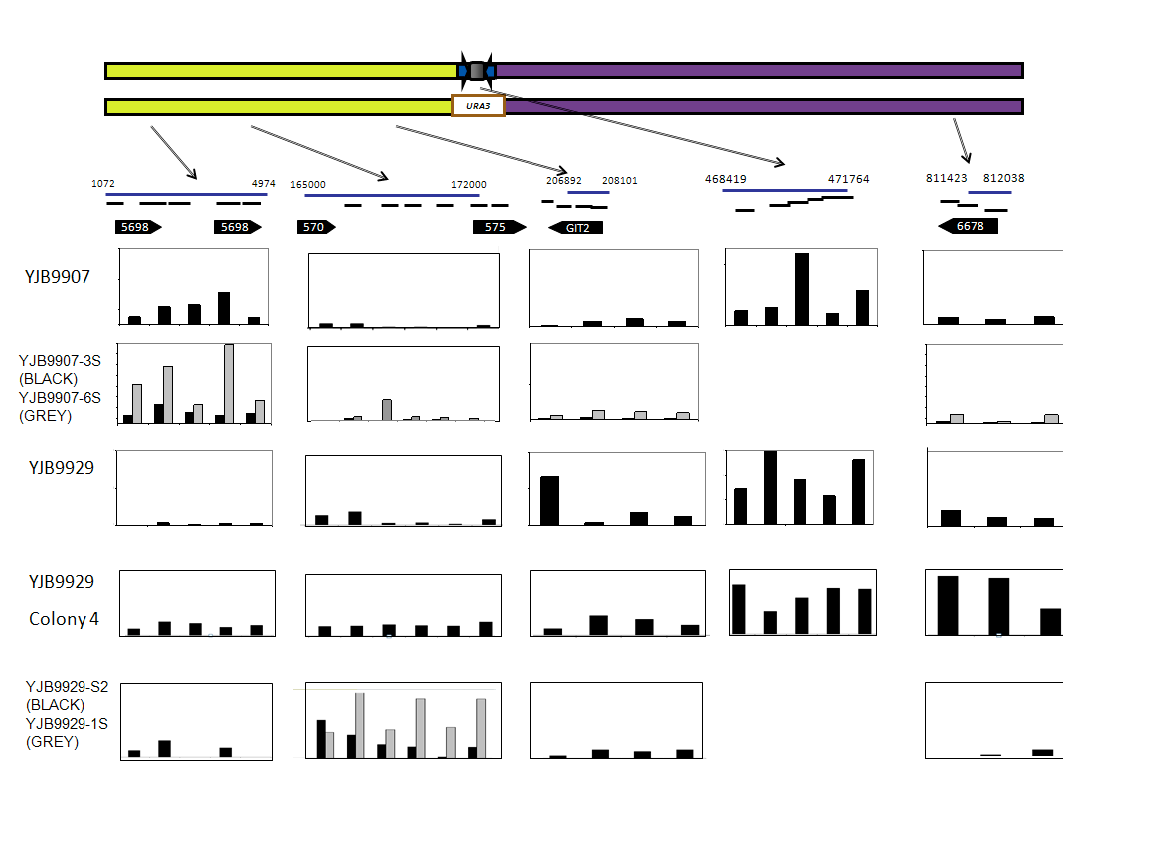

Supplement: Figure S4 — PCR analysis of neoCEN regions in Class B strains. Regions analyzed for strains indicated on left are within the peaks identified in Figure 8B. Chromosomal coordinates and positions of the PCR products relative to ORFs (black) arrows are diagramed and primer pairs used are listed in Table S1. A. Analysis of YJB9907 and its derivatives. Top, YJB9907; bottom, cen5Δ::URA3 homozygous strains YJB9907-3s (grey bars) and YJB9907-6s (black bars). No CEN5 DNA is present in these strains. B. Analysis of YJB9929 and its derivatives. Top panel, YJB9907; middle panel, single colony #4 from YJB9929 grown in rich medium; Bottom panel, cen5Δ::URA3 homozygous strains YJB9929-1s (grey bars) and YJB9929-2s (black bars). No CEN5 DNA is present in these strains. (0.1 MB TIF) [file pgen.1000400.s004.tif]
