# Supplementary material for: Neocentromeres Form Efficiently at Multiple Possible Loci in Candida albicans
Source: PLoS Genet. 2009 Mar 6;5(3):e1000400. doi: 10.1371/journal.pgen.1000400 (PMC2642679; doi:10.1371/journal.pgen.1000400)
Supplement: Table S1 — Strains used in this study. (0.1 MB DOC) [file pgen.1000400.s005.doc]

**Table S1. Strains used in this study**

**Strain number Strain features/names^1^ Relevant genotype Source**

**Parental strains**

YJB2348 SC5314 *MTLa/MTLalpha* prototroph [1,2]

YJB8653 CAI4 *SC5314 ura3::imm434/ura3::imm434* [2]

YJB8648 RM10 CAI4 *his1::hisG/HIS1* [3]

YJB7617 RM1000 RM10 *his1::hisG/his1::hisG* Chr5 not truncated [3]

YJB10064 marked Ch5R RM1000 *his1::hisG::HIS1/ his1::hisG::NAT1* This study

YJB3731 BWP17 RM1000 *arg4::hisG/arg4::hisG* [4]

***cen5∆* strains**

YJB9861 Long Distal/Class B RM10 *cen5∆::URA3/CEN5* This study

YJB9862 Short Distal/Class B RM10 *cen5∆::URA3/CEN5* This study

YJB9907 Short Distal/Class B RM10 *cen5∆::URA3/CEN5* This study

YJB9909 Short Proximal/Class A RM10 *cen5∆::URA3/CEN5* This study

YJB9915 Short Proximal/Class A RM10 *cen5∆::URA3/CEN5* This study

YJB9916 Long Proximal/Class A RM10 *cen5∆::URA3/CEN5* This study

YJB9926 Long Proximal/Class A RM10 *cen5∆::URA3/CEN5* This study

YJB9929 Long Distal/Class B RM10 *cen5∆::URA3/CEN5* This study

YJB10233 marked Ch5R Class A YJB10064 *cen5∆::URA3* This study

YJB10234 marked Ch5R Class B YJB10064 *cen5∆::URA3* This study

YJB10777 YJB9907s-3s Chr5 homozyg. YJB9907 *cen5∆::URA3/ cen5∆::URA3 MTLa/MTLa* This study

YJB10778 YJB9907-6ss Chr5 homozyg. YJB9907 *cen5∆::URA3/ cen5∆::URA3 MTLa/MTLa* This study

YJB10779 YJB9929s-1s Chr5 homozyg. YJB9929 *cen5∆::URA3/ cen5∆::URA3* *MTLa/MTLa* This study

YJB10780 YJB9929s-2s Chr5 homozyg. YJB9929 *cen5∆::URA3/ cen5∆::URA3 MTLa/MTLa* This study

YJB10805 Long RM10 *cen5D::NAT1/CEN5* This study

YJB10828 Short RM10 *cen5D::NAT1/CEN5* This study

**Other transformants/isolates**

YJB9955 BWP17 prototroph BWP17 *HIS1::his1 ARG4-URA3::arg4* [5]

YJB8287 I5(R) YJB6284 i(5R) [6]

YJB9779 5L-*URA3*-Tel RM1000 *Orf19.4216::URA3-TEL* This study

YJB9858 Tel-*NAT1*-5R RM1000 *Orf19.3161::NAT1-TEL*  This study

YJB9891 I5(L) clinical isolate(CHUV) [6]

YJB9984 *myo1∆::URA3* RM10 *myo1∆::URA3* This study

YJB10169 marked Ch5R *myo1∆::URA3* YJB10064 *myo1∆::URA3* This study

YJB8284 YJB9955s *Chr5* homozyg. YJB9955 *MTLa/MTLa* Chr5 homozygous This study

YJB8288 YJB9955s *Chr5* homozyg. YJB9955 *MTLa/MTLa* Chr5 homozygous This study

^1^ Short and long refer to the *CEN5* allele that was disrupted. The Long allele contains two LTRs absent in the short allele (See Fig. 1 and Fig. S1).

**Sources:**

1. Gillum AM, Tsay EY, Kirsch DR (1984) Isolation of the *Candida albicans* gene for orotidine-5'-phosphate decarboxylase by complementation of *S. cerevisiae* ura3 and E. coli pyrF mutations. Mol Gen Genet 198: 179-182.

2. Fonzi WA, Irwin MY (1993) Isogenic strain construction and gene mapping in *Candida albicans*. Genetics 134: 717-728.

3. Alonso-Monge R, Navarro-Garcia F, Roman E, Negredo AI, Eisman B, et al. (2003) The Hog1 mitogen-activated protein kinase is essential in the oxidative stress response and chlamydospore formation in *Candida albicans*. Eukaryot Cell 2: 351-361.

4. Wilson RB, Davis D, Mitchell AP (1999) Rapid hypothesis testing with *Candida albicans* through gene disruption with short homology regions. J Bacteriol 181: 1868-1874.

5. Bensen ES, Filler SG, Berman J (2002) A forkhead transcription factor is important for true hyphal as well as yeast morphogenesis in *Candida albicans*. Eukaryot Cell 1: 77-98.

6. Selmecki A, Forche A, Berman J (2006) Aneuploidy and isochromosome formation in drug-resistant *Candida albicans*. Science 313: 367-370.
