# Supplementary material for: Neocentromeres Form Efficiently at Multiple Possible Loci in Candida albicans
Source: PLoS Genet. 2009 Mar 6;5(3):e1000400. doi: 10.1371/journal.pgen.1000400 (PMC2642679; doi:10.1371/journal.pgen.1000400)
Supplement: Table S2 — Primers used in this study. (0.2 MB DOC) [file pgen.1000400.s006.doc]

**Supplementary material:**

Table S2. Primers used in this study

| **Forward primer #** | **Sequence** | **Reverse primer #** | **Sequence** | **Purpose** |  |
| --- | --- | --- | --- | --- | --- |
|  | **Primers for strain construction** |  |  |  |  |
| 2211 | CAATTGTAACTACAAATTGTTACAGTTTTCAAATTCTACTCAAACAAGCATTCGAAGGACATTAATTAACGTTTTCCCAGTCACGACGTT | 2212 | CTGACTCCTCTAATTAATCAATTCTACAAACTTGAATTCAAACTTTTTATTCCAGTATTCTGATTGATCTGTGGAATTGTGAGCGGATA | *cen5::URA3* disruption |  |
| 2002 | GATCACTGGTCTGTCAAC | 945 | CAAACAATCCTCTACCAACA | Verify *cen5::URA3* (5' end) |  |
| 1915 | CAGCCTTTGAGCCCTATT | 944 | AGACCTATAGTGAGAGAGCA | Verify *cen5::URA3* (3' end) |  |
| 2002 | GATCACTGGTCTGTCAAC | 1961 | TCTACTAAAATCAACGAAGC | WT *CEN5* (5' end) |  |
| 1901 | GGGATGAAGACTATGATGTGC | 1915 | CAGCCTTTGAGCCCTATT | WT *CEN5* (3' end) |  |
| 3161 | CAATTGTAACTACAAATTGTTACAGTTTTCAAATTCTACTCAAACAAGCATTCGAAGGACATTAATTAACAGACCTATAGTGAGAGAGCA | 3162 | TGACTCCTCTAATTAATCAATTCTACAAACTTGAATTCAAACTTTTTATTCCAGTATTCTGATTGATCGTAAAACGACGGCCAGTGAATTC | *cen5::NAT1* disruption |  |
| 2002 | GATCACTGGTCTGTCAAC | 1997 | TTCCGCGGCCGCTATGGCCGACGTCGACAAGCTTGCCTCGTCCCCGCCGGGTC | Verify *cen5::NAT1* disruption (5' end) |  |
| 2040 | GCATCACCTGGAACAGAAGTTCTGTATC | 1915 | CAGCCTTTGAGCCCTATT | Verify *cen5::NAT1* disruption (3' end) |  |
| 2249 | CAGGTGCGTCAACAAGTTATCTATTACCCTAAGAAACTAGGTAACGCTTATCCTCGCAAATATTACTATTTCTAGAAGGACCACCTTTGATTG | 1489 | GCAAACTCACCATTACCGG | Chr5 right arm truncation |  |
| 2102 | CCAGTAATAGTCCAGCAATTG | 944 | AGACCTATAGTGAGAGAGCA | Verify Chr5R truncation |  |
| 2313 | GATTAAGAGTTCTAAAATGAGTGTTCAAATTCATTAGTCGCGGATACCAATTAGAAGATGGTAATAGCCTCGTTTTCCCAGTCACGACGTT | 1489 | GCAAACTCACCATTACCGG | Chr5 left arm truncation |  |
| 2319 | AGATAATCGCCCGACTG | 2040 | GCATCACCTGGAACAGAAGTTCTGTATC | Verify Chr5L truncation |  |
| 2543 | CAGAGGAAGAAGACAGCGAAGGTATAGTCAAAAAGCAATAGGACAGCAAGACAATTTATTTATTTAATAGAGACCTATAGTGAGAGAGCA | 2544 | CGTAATAATCAAAATGAGTCAATTCTCACAACCGCTCGCGACACGTTTCAACGAAATGGCCTCCCCTACCGTAAAACGACGGCCAGTGAATTC | *his1::NAT1* disruption |  |
| 728 | GTGTCGCCAGAATGTGCCGTTGTGTTTGTC | 565 | CTCGAGTACCAATATATCGGTTGC | Verify *his1::NAT1* disruption |  |
| 944 | AGACCTATAGTGAGAGAGCA | 945 | CAAACAATCCTCTACCAACA | Verify *URA3* integration |  |
| 1193 | TTGAAGCGTGAGAGGCAGGAG | 1194 | GTTTGGGTTCCTTCTTTCTCATTC | *MTLa* |  |
| 1195 | TTCGAGTACATTCTGGTCGCG | 1197 | TGTAAACATCCTCAATTGTACCCGA | MTLa |  |
|  |  |  |  |  |  |
|  | **Primers for ChIP detection** |  |  |  | **Median Chromosome Coordinate** |
| 2082 | TCCAAAACCATGCGGAAT | 2083 | TGGCAAATTTACAGCCGAA | *TAC1* | 419133 |
| 2922 | ACCAGAGCAGTGCATCAACCATC | 2923 | CTTCTACGAAGGGTAACAGG | *CEN4* | 992682 |
| 2924 | CCTGTTACCCTTCGTAGAAG | 2925 | GATGGTAGAGTTCCTCCTGC | *CEN4* | 993137 |
| 2926 | GCAGGAGGAACTCTACCATC | 2927 | CGCCATGAGAAATCAGGGAT | *CEN4* | 993539 |
| 2375 | ATCCCTGATTTCTCATGGCG | 2376 | ATGCCCTTGAATCTGTGACG | *CEN4* | 993923 |
| 2928 | CGTCACAGATTCAAGGGCAT | 2929 | TTGCCACTAAGTGGACCCAT | *CEN4* | 994314 |
| 2930 | ATGGGTCCACTTAGTGGCAA | 2931 | GGAAGTTTCGTCAGTAACTAGC | *CEN4* | 994779 |
| 2932 | GCTAGTTACTGACGAAACTTCC | 2933 | GTCAAACCTAGCACGCTAGT | CEN4 | 995225 |
| 2439 | GGTGCCAGCACAAAGTATTG | 2440 | AACAAGCATCTGCCAGACCT | *CEN6* | 980062 |
| 2848 | GCTGTTAACACAGTTAAGGC | 2847 | GGACTACTTCTCACAGTTCG | Left flank *CEN5* | 463430 |
| 2846 | CGAACTGTGAGAAGTAGTCC | 2845 | ATGAAGTTCCTCCAACGTTG | Left flank *CEN5* | 463850 |
| 2844 | CAACGTTGGAGGAACTTCAT | 2843 | CGAGTTTTGGTTACTCTACC | Left flank *CEN5* | 464275 |
| 2842 | GGTAGAGTAACCAAAACTCG | 2841 | TGTGCTGCGACGTTGAAT | Left flank *CEN5* | 464704 |
| 2911 | ATTCAACGTCGCAGCACA | 3027 | ACTCCTCTGAAACCTGACAA | Left flank *CEN5* | 465132 |
| 3026 | GCTCATCTTTATCCTCTTCG | 2838 | ACCTGACAAAGGCGGTGGA | Left flank *CEN5* | 465562 |
| 2837 | TCCACCGCCTTTGTCAGGT | 2836 | AGATAATCGCCCGACTG | Left flank *CEN5* | 465990 |
| 2003 | CAGTCGGGCGATTATCT | 3123 | CCTTCGAATGCTTGTTTGAG | LTR | Left LTR |
| 2002 | GATCACTGGTCTGTCAAC | 324 | CTTCAACTGTAAAAGGGGCG | *URA3-CEN5* junction | 5' region of *URA3* |
| 1899 | TTTGGCACCTTCGGTAGC | 2895 | GTCAATTTCTCTAGTAGAGGT | *CEN5* inverted repeat | 472910/468132 |
| 2894 | ACCTCTACTAGAGAAATTGAC | 2867 | GTAATAGAGAGCCAGAATCC | *CEN5* inverted repeat | 473509/467740 |
| 2868 | GGATTCTGGCTCTCTATTAC | 2892 | TGAAATGAACTTGCAATGTT | *CEN5* inverted repeat | 473061/467233 |
| 2891 | AACATTGCAAGTTCATTTCA | 2893 | GCACAAGAGGTTCAGCCGTGACC | *CEN5* inverted repeat | 472580/466807 |
| 1900 | GGTCACGGCTGAACCTCTTGTGC | 3025 | GTAGCCATAGTGTGGAGCTA | *CEN5* inverted repeat | 472180/466404 |
| 2898 | GGCTAAACAGCCATACCTCT | 2901 | TATCAGAAGCGGTGTTTGAG | *CEN5* central core | 468820 |
| 2900 | CTCAAACACCGCTTCTGATA | 2869 | TGGCCGCAACGTAATCTATG | *CEN5* central core | 469464 |
| 3023 | CTTCACAAGTGTCGTATAGC | 3024 | CAAAATAGCCTTGTTGTTGT | *CEN5* central core LTR | CC LTR |
| 2870 | ACGGTCCCTACGTTCGTCAA | 2871 | AGCCTCGTTGACCGGAAGTA | *CEN5* central core | 470307 |
| 2010 | TACTTCTGGTCAACGAGGCT | 2873 | CCAATACAGGTTCCAATATG | *CEN5* central core | 470729 |
| 2872 | CATATTGGAACCTGTATTGG | 2011 | GACTGACATCCGTACTATCG | *CEN5* central core | 471093 |
| 2896 | CGATAGTACGGATGTCAGTC | 2897 | CCACACTATGGCTACCACAA | *CEN5* central core | 471779 |
| 725 | GAAGGTAAAAGGTATAGAAATGCTGGTTGG | 1915 | CAGCCTTTGAGCCCTATT | *URA3* | 3' region of *URA3* |
| 3126 | GGATTTATTGCCCAACGTG | 3124 | TTCCAGTATTCTGATTGATC | *URA3* | 3' region of *URA3* |
| 2851 | AATAGGGCTCAAAGGCTG | 2852 | AAAGAGGCAGGGGTCTGG | Right flank *CEN5* | 474633 |
| 1910 | CCAGACCCCTGCCTCTTT | 2854 | CTAACGAATCCCGCGCAAAC | Right flank *CEN5* | 475134 |
| 2853 | GTTTGCGCGGGATTCGTTAG | 2856 | GGTATAAGTGCTATTAAGCG | Right flank *CEN5* | 475616 |
| 2855 | CGCTTAATAGCACTTATACC | 2858 | GCCAAACAGATTGCTCCTTG | Right flank *CEN5* | 476062 |
| 2857 | CAAGGAGCAATCTGTTTGGC | 2860 | GTGAATCTGATGGTGTAAAC | Right flank *CEN5* | 476481 |
| 2859 | GTTTACACCATCAGATTCAC | 2862 | CGATGTCAAAGAGTAGAAC | Right flank *CEN5* | 476904 |
| 2861 | GTTCTACTCTTTGACATCG | 2864 | GTGGAGCTGTACTAAATTGG | Right flank *CEN5* | 477328 |
| 2863 | CCAATTTAGTACAGCTCCAC | 2866 | TACACGTTTGTTCGTCAGAG | Right flank *CEN5* | 477753 |
| 3247 | TTTCTCTTCACCTGTCGTTACCG | 3248 | GGGCAACTACATCTACAAGGACCG | neoCEN-1, intergenic | 1257 |
| 3249 | ACACCTTTATTTCCCCCCACC | 3250 | CTTCTTTGCTGCTTCTTCTGCC | neoCEN-1, TLO11 | 2228 |
| 3251 | AAGCAGCAAAGAAGGCAGAGACCG | 3252 | CCACCAAAAAATAACCACCTCAGG | neoCEN-1, intergenic | 2812 |
| 3253 | GCAATGGTGCTTGGTCAACTCTC | 3254 | GGTGGTGAAGACGACTGGTTTG | neoCEN-1, orf19.5698 | 4062 |
| 3255 | TTTTGGCGGCAGAGAGTTGC | 3256 | ATCGTTGTTTTGGGGTGGTTC | neoCEN-1, orf19.5698 | 4659 |
| 3271 | ATCGGAGCAGTTCGCCTATT | 3272 | GTTTAGCCCATCCAAAGGCA | *neoCEN-2, intergenic* | 206113 |
| 3269 | TCTTGCCTTTGGATGGGCTA | 3270 | TCCGAGGCAATGACAGAAGA | *neoCEN-2, GIT2* | 206638 |
| 3273 | GCCATTCTTCCAACTGCCTT | 3274 | TACCGCCAGGACCAAATTCA | *neoCEN-2, GIT2* | 207301 |
| 3275 | TGTCAGCTTGTTTGAACGGG | 3276 | AGAACTGCTGTCTTCTCCCA | *neoCEN-2, GIT2* | 207658 |
| 3336 | ACGAACAACAATAGACGGAGCG | 3337 | TGCTTTCCACGAGCCAATAAC | neoCEN-3, orf19.6678 | 810477 |
| 3338 | GCATTTATCATTTACTTGAAGAGCCCC | 3339 | CACAAAAACAGAATAGCCTGGATG | neoCEN-3, orf19.6678 | 811111 |
| 3340 | GGCTATTCTGTTTTTGTGATTACCG | 3341 | GGCTTTACCCCTTTTTTCTCTTC | neoCEN-3, orf19.6678 | 811524 |
| 3192 | CTGGTGAAGTGATTCCAAATGC | 3193 | CCAATGAACGCAATGAACACAG | neoCEN-4, intergenic | 166890 |
| 3194 | TGGCATCACAACATAAGC | 3195 | TGAGAGAAGGAGAAAAATCAGCG | neoCEN-4, intergenic | 168691 |
| 3163 | CGTTGTTGGTGTCCTTTTCCC | 3164 | TGCGAATACATTTTTCCACTGC | neoCEN-4, intergenic | 169656 |
| 3196 | CGTTGTTGGTGTCCTTTTCCC | 3197 | TGCGAATACATTTTTCCACTGC | neoCEN-4, orf19.575 | 171293 |
| 3198 | TGACACAGGGATTTTGACGGTG | 3199 | GGGAAAGCAGAAGGTTCAGTGG | neoCEN-4, orf19.575 | 172059 |
| 3200 | AATCTGGAAGCGAATCCGTTG | 3201 | TTCACTGGCTGATGATGGTGC | neoCEN-4, orf19.575 | 172583 |
